# Sp3 is essential for normal lung morphogenesis and cell cycle progression during mouse embryonic development

**DOI:** 10.1242/dev.200839

**Published:** 2023-03-03

**Authors:** Alyssa M. McCoy, Omar Lakhdari, Sayane Shome, Kaitlin Caoili, Gilberto E. Hernandez, Nima Aghaeepour, Lindsay D. Butcher, Kathleen Fisch, Lawrence S. Prince

**Affiliations:** ^1^ Department of Pediatrics, University of California, San Diego, La Jolla, CA 92093, USA; ^2^ Department of Pharmacology, Meharry Medical College, Nashville, TN 37208, USA; ^3^ Department of Pediatrics, Stanford University, Palo Alto, CA 94304, USA; ^4^ Department of Anesthesiology, Perioperative and Pain Management, Stanford University, Palo Alto, CA 94305, USA; ^5^ Department of Obstetrics, Gynecology, and Reproductive Services, University of California, San Diego, La Jolla, CA 92093, USA; ^6^ Center for Computational Biology & Bioinformatics, University of California, San Diego, La Jolla, CA 92093, USA

**Keywords:** Alveolar development, Transcriptional regulation, Cell proliferation, Lung mesenchyme, Lipofibroblast, Myofibroblast

## Abstract

Members of the Sp family of transcription factors regulate gene expression via binding GC boxes within promoter regions. Unlike Sp1, which stimulates transcription, the closely related Sp3 can either repress or activate gene expression and is required for perinatal survival in mice. Here, we use RNA-seq and cellular phenotyping to show how Sp3 regulates murine fetal cell differentiation and proliferation. Homozygous *Sp3^−/−^* mice were smaller than wild-type and *Sp^+/−^* littermates, died soon after birth and had abnormal lung morphogenesis. RNA-seq of *Sp3^−/−^* fetal lung mesenchymal cells identified alterations in extracellular matrix production, developmental signaling pathways and myofibroblast/lipofibroblast differentiation. The lungs of *Sp3^−/−^* mice contained multiple structural defects, with abnormal endothelial cell morphology, lack of elastic fiber formation, and accumulation of lipid droplets within mesenchymal lipofibroblasts. *Sp3^−/−^* cells and mice also displayed cell cycle arrest, with accumulation in G_0_/G_1_ and reduced expression of numerous cell cycle regulators including *Ccne1*. These data detail the global impact of Sp3 on *in vivo* mouse gene expression and development.

## Teaser

**Summary:** The ubiquitous transcription factor Sp3 is required for the development of gas exchange regions of the lung in addition to controlling expression of multiple cell cycle regulators.

## INTRODUCTION

Throughout the genome, transcription factor binding facilitates chromatin unwinding and marks genes for mRNA transcription. Complex regulation of transcription factor binding, and subsequent gene expression, drives diverse biological processes during development and homeostasis. The actual process of mRNA transcription relies on conserved molecular machinery binding to various promoter elements. In genes with promoters containing TATA box sequences, associations of TATA binding protein (TBP) with the pre-initiation complex drive conformational changes in the DNA structure and recruit RNA polymerase II to initiate transcription (Bhuiyan and Timmers, 2019; Lee and Hahn, 1995). In gene promoters lacking a TATA box, transcription initiation can begin at GC-rich regions known as GC boxes (classically 5′-GGGGCGGGG-3′) or GT/GACC boxes (5′-GGTGTGGGG-3′) (Gidoni et al., 1985). Formation of the pre-initiation complex at GC boxes requires DNA binding by members of the specificity protein (Sp) or Kruppel-like factor (KLF) family of transcription factors (Suske, 1999; Kaczynski et al., 2003). Sp/KLF members bind GC-rich regions via conserved tripartite zinc-finger DNA binding motifs and cooperate with TBP to facilitate pre-initiation complex formation. Understanding how specific factors promote gene transcription by these core mechanisms therefore provides important biological insight into developmental and disease processes.

GC boxes are widespread in the mammalian genome, with approximately a quarter of promoters containing at least one GC sequence (Suske, 2017). Mammals express nine Sp family members and 17 KLF transcription factors, each with conserved DNA binding domains (Kaczynski et al., 2003; Suske et al., 2005; Philipsen and Suske, 1999). Sp1 (originally named for the Sephacryl and Phosphocellulose columns used for its purification) activates gene transcription when bound to GC boxes, both within the promoter region and distant enhancers (Dynan and Tjian, 1983; O'Connor et al., 2016; Cawley et al., 2004; Pascal and Tjian, 1991). Like Sp1, the mammalian family member Sp3 binds GC sequences and is ubiquitously expressed. When a single GC box is present, Sp1 and Sp3 appear to have similar transcriptional activation functions (Udvadia et al., 1995). However, in genes with multiple GC sequences, Sp3 can either activate or repress transcription (Birnbaum et al., 1995; Dennig et al., 1996; Sjottem et al., 1996). The inhibitory function of Sp3 may require post-translational modifications and could involve interfering with higher order Sp1 complex formation or facilitating recruitment of other transcriptional repressors (Valin and Gill, 2013; Stielow et al., 2010; Sapetschnig et al., 2002; Ross et al., 2002). Co-expressed in most cells, Sp1 and Sp3 appear to localize to distinct nuclear regions (He et al., 2005). While sharing similar DNA-binding domains, Sp1 and Sp3 have distinct C-terminal regions that likely contribute to their unique functions (Suske, 1999).

Sp3 interacts with other regulatory proteins to impact chromatin accessibility and mRNA transcription (Li et al., 2004). Having the ability to function as both activator and repressor, Sp3 demonstrates complex biological functions in regulating genes important for normal cell biology, developmental processes and response to environmental stimuli. We previously demonstrated how Sp1 and Sp3 differentially regulate the key developmental gene *Fgf10*, including regulation of *Fgf10* promoter activity by interactions of Sp3 with the innate immune response transcription factor NF-κB (Carver et al., 2013). Knockout mice lacking Sp3 die at birth and display multiple developmental phenotypes. *Sp3^−/−^* embryos also had defective bone ossification and tooth development (Gollner et al., 2001; Bouwman et al., 2000). Definitive hematopoiesis was impaired in *Sp3^−/−^* embryos, with delayed production of erythrocytes and partial defects in lymphocyte development (Van Loo et al., 2003). Although the exact cause of perinatal mortality in *Sp3^−/−^* mice was unclear, abnormalities in lung development could contribute to reduced survival at birth (Bouwman et al., 2000).

Here, we present a comprehensive analysis of gene transcription to globally measure the impact of Sp3 on lung developmental biology and embryonal growth. We performed RNA-seq using fetal lung mesenchymal cells from *Sp3^−/−^* mice. In depth analysis of the gene pathways differentially expressed in Sp3 mutants identified several distinct biological areas regulated by Sp3. Cells lacking Sp3 failed to express many genes important for extracellular matrix assembly, with abnormalities in myofibroblast/lipofibroblast differentiation. We also identified a defect in cell cycle regulation in *Sp3^−/−^* cells, leading to failed proliferation in culture and likely contributing to the abnormal growth of Sp3 mutant embryos. These results give a genome-wide assessment of Sp3-dependent transcriptional mechanisms and identify a number of key biological processes regulated by this ubiquitous but complex transcription factor.

## RESULTS

### Sp3 is required for normal lung growth and distal airway morphogenesis

**Fig. 1.**
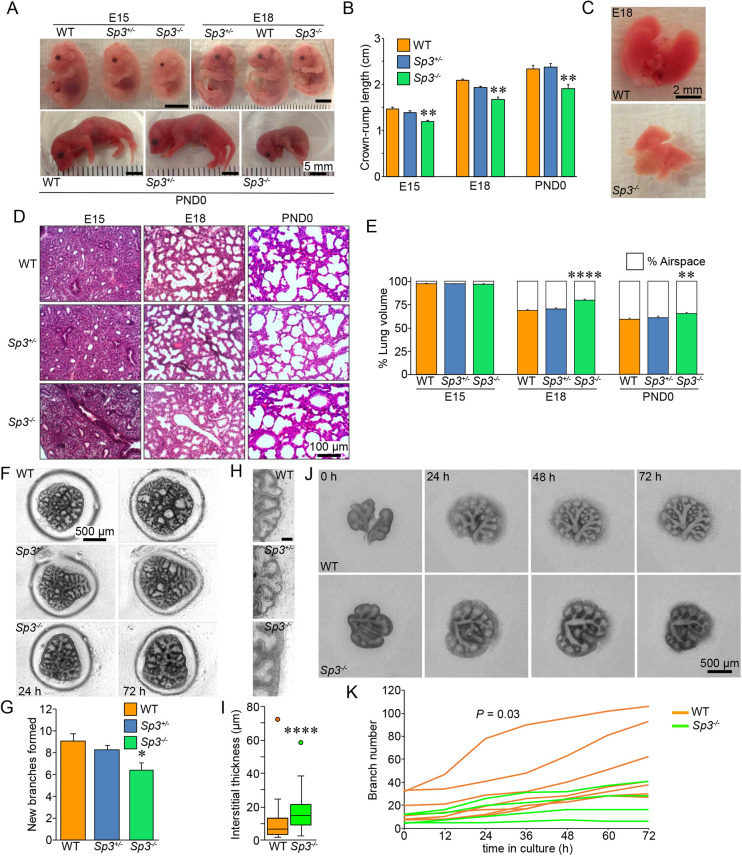
**Sp3 is required for normal lung growth and distal airway morphogenesis.** (A) Gross appearance of WT, *Sp3^+/−^* and *Sp3^−/−^* embryos (E15 and E18) and neonates (PND0). (B) *Sp3^−/−^* embryos and neonates had shorter crown-rump length compared with WT and *Sp3^+/−^* littermates. Data are expressed as mean±s.e.m., *n*=4-10 mice for each genotype. **P<0.01 compared with WT using two-tailed unpaired *t*-test. (C) Gross appearance of WT and *Sp3^+/−^* E18 hearts and lungs. (D,E) Hematoxylin and Eosin-stained lung sections from E15, E18 and PND0 WT, *Sp3^+/−^* and *Sp3^−/−^* mice (D). Representative sections from littermates are shown. Lung morphometry measurements showed reduced airspace in *Sp3^−/−^* lungs at E18 and PND0 (E). Data expressed as mean±s.e.m., *n*=3-5 mice for each genotype at each time point. Number of random images analyzed for each genotype: E15, 17-24; E18, 26-27; PND0, 47-50. **P<0.01, ****P<0.0001 compared with WT at each time point using two-tailed unpaired *t*-test. (F,G) Reduced saccular airway branch formation in *Sp3^−/−^* fetal lung explants (F). E15 fetal lung explants were cultured for 72 h with images acquired at 24 and 72 h. Brightfield images show new branch formation and changes in branch morphology along the periphery. *Sp3^−/−^* explants developed fewer airways compared with WT and *Sp3^+/−^* explants (G). Data expressed as mean±s.e.m. Explants were isolated from five separate litters, with 6-15 embryos per genotype; 39-106 explants analyzed. Representative images are shown. *P<0.05 versus WT using two-tailed unpaired *t*-test. (H,I) Increased interstitium in *Sp3^−/−^* E15 fetal lung explants. Images were obtained 24 h after culture and peripheral interstitial regions were measured using ImageJ. Representative images are shown in H. Scale bar: 20 μm. Box and whisker plots of measurements are shown in I. Plots show median values (middle bars) and first to third interquartile ranges (boxes); whiskers indicated 1.5 times the interquartile ranges; dots indicate outliers. ****P<0.001 versus WT using two-tailed unpaired *t*-test. WT, 96 measurements were included; *Sp3^−/−^*, 68 measurements. (J) Time lapse images of E12 lungs cultured for 72 h. (K) Total branch number for each E12 lung measured every 12 h. The number of new branches formed in each lung during every 12 h period in WT and *Sp3^−/−^* lungs was calculated and compared using the Wilcoxon signed rank test (*n*=42 for WT, 24 for *Sp3^−/−^*; *P*=0.03).

To better understand the role of Sp3 in murine development, we examined litters of fetal mice lacking one or both copies of the *Sp3* allele (Fig. 1). As homozygous *Sp3^−/−^* mice are nonviable, timed mating of hemizygous *Sp3^+/−^* mice were used to generate mutant and wild-type (WT) embryos as well as neonates. Crown-rump measurements demonstrated that at embryonic day (E)15 and E18, *Sp3^−/−^* mice were smaller than WT and *Sp3^+/−^* littermates (Fig. 1A), consistent with previous reports (Bouwman et al., 2000). Live video monitoring of deliveries allowed immediate measurement of postnatal day (PND)0 *Sp3^−/−^* pups, who continued to have shorter crown-rump lengths than littermate controls (Fig. 1B). Additional defects in gross morphology were not observed upon examination.

PND0 *Sp3^−/−^* mice were observed gasping with respiratory distress. Although their rapid postnatal deaths precluded additional physiological assessments, the phenotype suggested neonatal death in mice lacking Sp3 could be due to defects in lung development. We therefore examined structural lung morphology in embryonal and neonatal Sp3 mutants. The lungs of *Sp3^−/−^* embryos were grossly normal but smaller than WT and *Sp3^+/−^* littermates (Fig. 1C). Histological sectioning and airway morphometry measurements (Fig. 1D,E) showed that pseudoglandular stage development at E15 was similar in embryos of all genotypes. However, saccular airway development in *Sp3^−/−^* lungs appeared to be abnormal at E18. Lungs from E18 *Sp3^−/−^* mice had reduced airspace volume percent compared with WT and *Sp3^+/−^* littermates (****P<0.0001, two-tailed unpaired *t*-test). This defect in distal airway formation persisted in PND0 *Sp3^−/−^* mice. We also studied saccular stage airway morphogenesis in *Sp3^−/−^* lungs using E15 fetal lung explants. Fig. 1F,G shows increased airway branching in WT and *Sp^+/−^* explants over 48 h, with fewer new branches formed in *Sp3^−/−^* explants. *Sp3^−/−^* lungs and explants displayed thicker interstitial regions, consistent with the reduced saccular airspace in E18 and PND0 *Sp3^−/−^* mice (Fig. 1H,I). To test whether Sp3 was also required for earlier airway branching, we isolated lungs from E12 WT and *Sp3^−/−^* litters, measuring total airway branches upon isolation and then every 12 h during *ex vivo* culture via live cell microscopy (Fig. 1J). Although we did not measure significant differences in the number of early airways at E12, *Sp3^−/−^* lungs acquired fewer new branches over time compared with WT lungs (Fig. 1K). These data showed that Sp3 was therefore required for normal airway morphogenesis, possibly contributing to neonatal mortality.

### Differential gene expression in *Sp3^−/−^* fetal lung mesenchymal cells

**Fig. 2.**
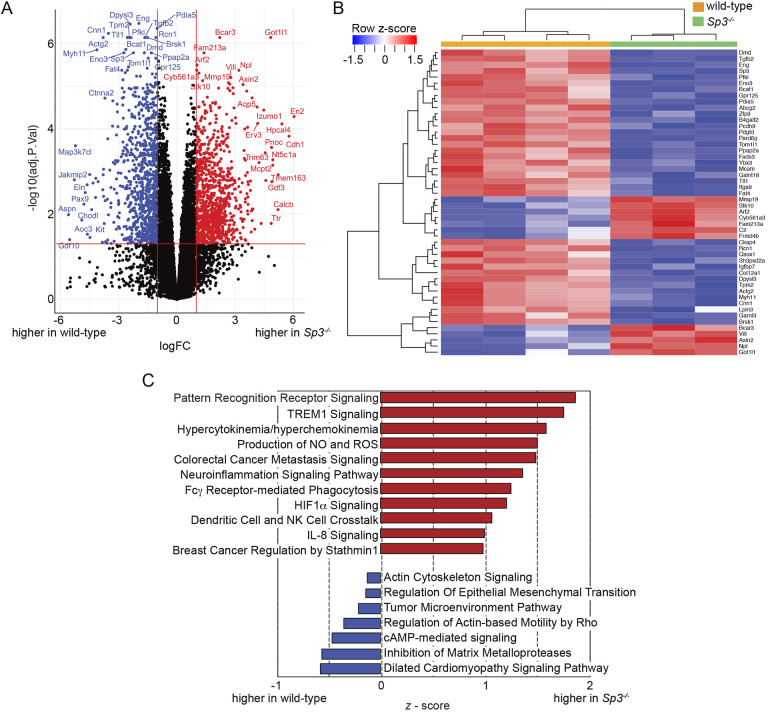
**Differential gene expression in *Sp3^−/−^* fetal lung mesenchymal cells.** (A) Volcano plot showing differentially expressed genes in WT and *Sp3^−/−^* fetal lung mesenchymal cells. Red, genes upregulated in *Sp3^−/−^* cells (adjusted *P*-value <0.05 and |log_2_FoldChange|>1); blue, genes downregulated in *Sp3^−/−^* cells (adjusted *P*-value <0.05 and |log_2_FoldChange|>1). (B) Unsupervised hierarchically clustered heatmap with normalized expression (*z*-score) of 50 most differentially expressed genes when comparing WT with *Sp3^−/−^* RNA-seq datasets. Data for each replicate are shown (*n*=3 for *Sp3^−/−^* cells, *n*=4 for WT cells). Raw count data was transformed to log_2_-counts per million followed by TMM-normalization before differential expression analysis using the limma-voom method. (C) IPA of differentially expressed genes. Categories of transcripts more enriched in *Sp3^−/−^* cells are shown in red with a *z*-score>0. Categories higher in WT cells are shown in blue with *z*-score<0.

**Fig. 3.**
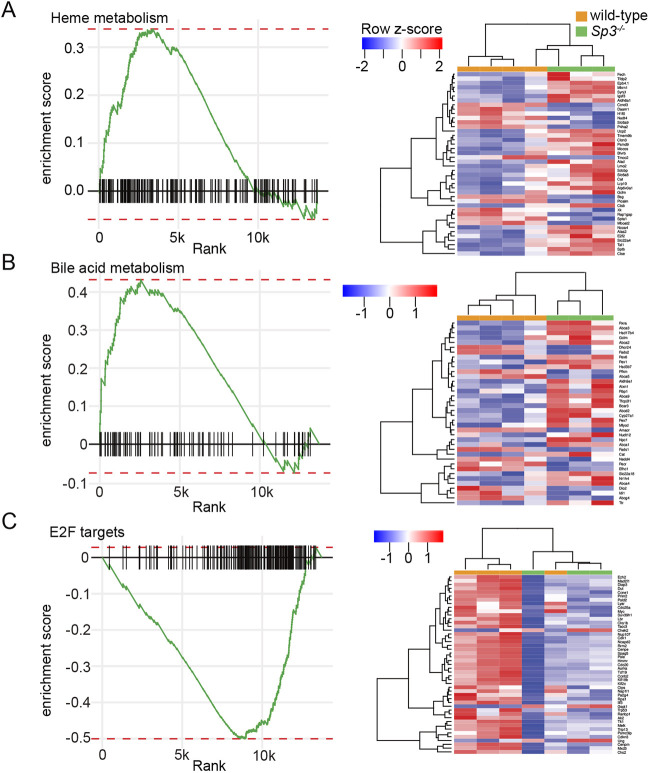
**GSEA analysis of differentially expressed genes in WT and *Sp3^−/−^* cells.** (A-C) GSEA of differentially expressed transcripts in *Sp3^−/−^* and WT cells revealed enriched gene sets related to heme metabolism (A), bile acid metabolism (B) and E2F targets (C). Heat maps showing normalized expression of leading-edge transcripts shown in right panels for each GSEA category. *n*=3 for *Sp3^−/−^*, *n*=4 for WT.

**Fig. 4.**
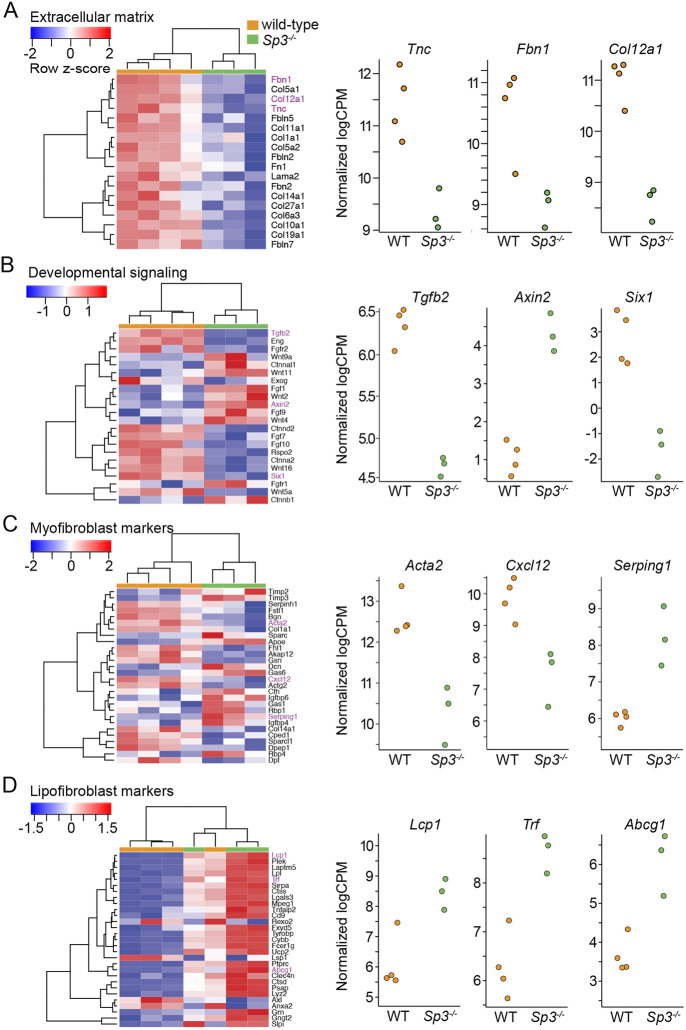
**Impact of Sp3 deletion on specific mesenchymal gene categories.** (A-D) Lists of transcripts related to extracellular matrix (A), developmental signaling (B), myofibroblast markers (C) and lipofibroblast markers (D) were analyzed in WT and *Sp3^−/−^* samples. Heat maps of differentially expressed genes within each category are shown in left panels. Transcripts expressed at higher normalized levels are indicated in red and lower levels in blue. For select genes, individual normalized CPM for each replicate are shown in right panels. *n*=3 for *Sp3^−/−^*, *n*=4 for WT.

Lung branching morphogenesis involves precise epithelial-mesenchymal cell interactions. Our previous data (Carver et al., 2013) and recent single cell analyses detected Sp3 expression in all cell populations throughout the developing lung (Fig. S1). We did not detect significant differences in the expression of the epithelial transcripts *Sox2*, *Sox9*, *Shh, Bmp4, Fgfr2* or *Sftpc* in WT and *Sp3^−/−^* total lung RNA using real time PCR (Fig. S2). To next focus on potential Sp3-dependent differences in the fetal lung mesenchyme, we isolated primary mesenchymal cells from E15 Sp3 mutant and WT lungs for analysis. After a brief culture period, we compared the transcriptional profiles of WT and *Sp3^−/−^* fetal lung mesenchymal cells by RNA-seq. A volcano plot (Fig. 2A) illustrates the differences in gene expression between WT and *Sp3^−/−^* fetal lung mesenchymal cells along with noting selected genes. The heat map in Fig. 2B shows the normalized expression of the 50 most differentially expressed genes in each sample based on genotype. Ingenuity Pathway Analysis (IPA) of all differentially expressed genes identified potential Sp3-dependent roles in multiple developmental categories (Fig. 2C). The reduced expression in *Sp3^−/−^* cells of genes related to the actin cytoskeleton, epithelial-mesenchymal transition and matrix metalloproteinases could lead to abnormal lung morphogenesis. Gene set enrichment analysis (GSEA) identified several distinct groups of genes, the expression of which was impacted by loss of Sp3 (Fig. 3). The normalized enrichment scores (NES) were higher in *Sp3^−/−^* cells for genes related to heme and bile acid metabolism, whereas they were lower for genes related to E2F regulation of the cell cycle. Inspection of differentially expressed genes from *Sp3^−/−^* mesenchymal cells identified multiple targets involved in formation of the extracellular matrix, signaling events during lung morphogenesis and markers of lung lipofibroblast and myofibroblast phenotypes (Fig. 4). *Tnc* and *Fbn1* are both key components of the extracellular matrix during lung morphogenesis and are lower in *Sp3^−/−^* cells (Mecham, 2018; Young et al., 1994; Gremlich et al., 2020; Fig. 4A). The differential expression of *Tgfb2* and *Axin2* (Fig. 4B) was interesting as we did not detect differences in WT and *Sp3^−/−^* total lung RNA (Figs S3, S4). Although using total RNA from the dozens of cells within the lung limits resolution, isolating mesenchymal cells for even brief culture times could also change cellular transcription.

Along with the decreased expression of multiple extracellular matrix components, *Sp3^−/−^* mesenchymal cells also had altered expression of myofibroblast markers, with reduced counts of *Acta2* and *Cxcl12* (Fig. 4C). However, *Serping1* was higher in *Sp3^−/−^* mesenchymal cells and inspection of the myofibroblast marker heat map revealed that loss of Sp3 did not produce uniform increase or decrease in all genes. In comparison, lipofibroblast markers were more likely to be expressed at higher levels in *Sp3^−/−^* cells (Fig. 4D). These complementary analyses each suggested the requirement of Sp3 in regulating lung mesenchymal phenotype and function.

### Sp3 is required for normal vascular development in mouse lungs

**Fig. 5.**
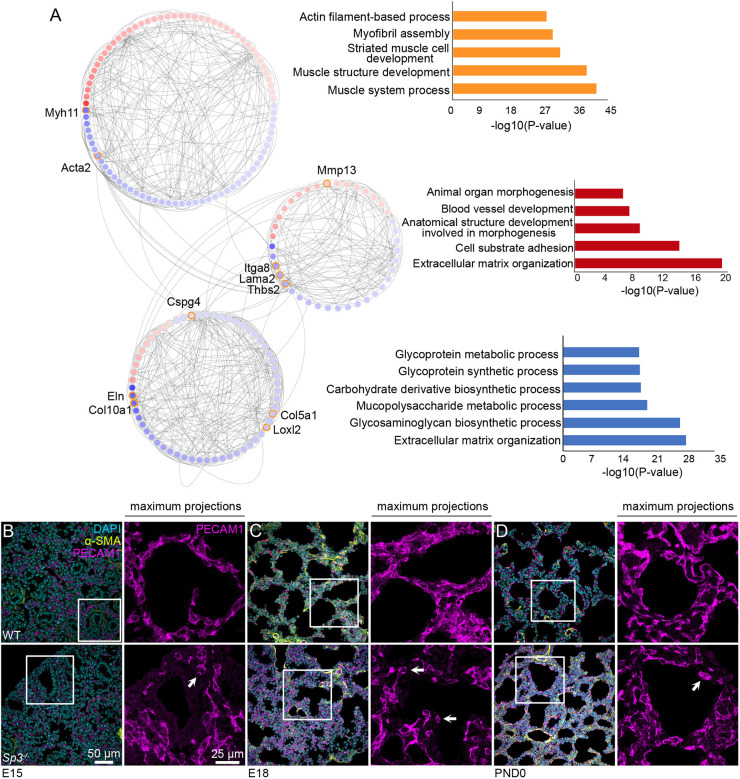
**Sp3 is required for normal vascular development in mouse lungs.** (A) Three adjacent subnetworks identified from network analysis of *Sp3^−/−^* and WT differentially expressed genes were identified with biological properties related to cell-matrix interactions, tissue structure, and blood vessel development. GO terms of differentially expressed node members are shown to the right. (B-D) Altered endothelial cell morphology in *Sp3^−/−^* lungs at E15 (B), E18 (C) and PND0 (D). Sections labeled with DAPI (cyan), anti-α-SMA (yellow) and anti-PECAM1 (magenta) and imaged by confocal microscopy. Maximum *z*-projections of anti-PECAM1 labeling corresponding to the area outlined in white are shown at the right of each panel. White arrows indicate endothelial cells with rounder morphology in *Sp3^−/−^* lungs. Images shown are representative of samples from three separate litters.

To better characterize the multiple biological processes regulated by Sp3, we interrogated the network neighborhood of differentially expressed genes to identify subnetworks of closely interconnected genes. Fig. 5A shows three subnetworks and their enriched GO terms. These connections and patterns suggest that Sp3 is required for normal transcription of genes related to contractile phenotypes and extracellular matrix organization. Several noteworthy genes, including *Acta2*, *Myh11*, *Col10a1*, *Col5a1*, *Eln*, *Loxl2*, *Mmp13*, *Thbs2* and *Lama2* are highlighted in Fig. 5. In addition, expression of the mesenchymal integrin *Itga8* and the pericyte marker *Cspg4* were both reduced in *Sp3^−/−^* mesenchymal cells. The reduced expression of genes related to mechanical properties, interactions with the extracellular matrix, and pericyte identity prompted examination of the fetal lung vasculature in *Sp3^−/−^* lungs (Fig. 5B-D). The lung capillary network normally undergoes dramatic expansion and remodeling during the pseudoglandular and saccular stages of lung development. In WT E15 lungs, PECAM1^+^ endothelial cells within the vascular plexus extended cellular processes surrounding the developing airways (Fig. 5B). Endothelial cells in *Sp3^−/−^* E15 lungs appeared to be less connected with rounder morphology. This difference was also observed at E18 (Fig. 5C). At PND0, the differences in endothelial cell morphology between WT and *Sp3^−/−^* lungs were still present although less obvious (Fig. 5D).

### Sp3 regulates lung mesenchymal differentiation and phenotype

**Fig. 6.**
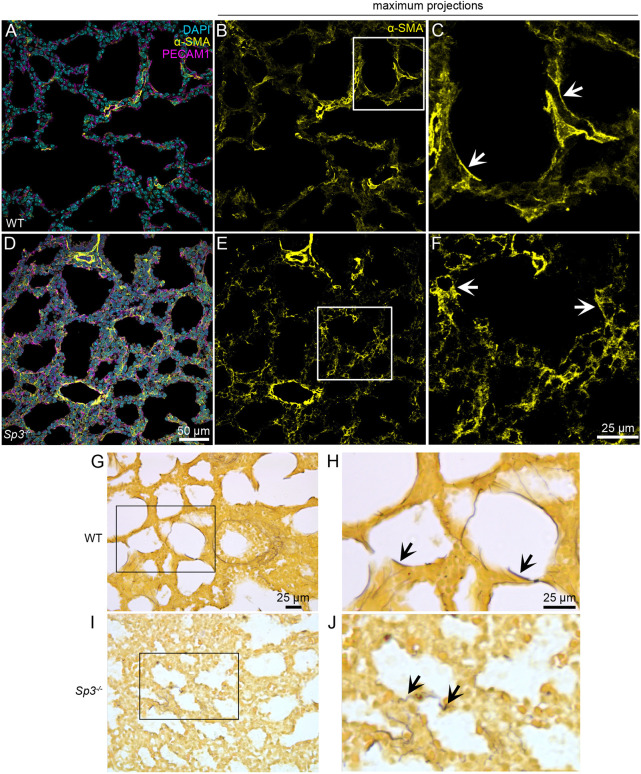
**Disruption of myofibroblast morphology and elastic fibers in *Sp3^−/−^* mouse lungs.** (A-F) Lung sections from PND0 WT (A-C) and *Sp3^−/−^* (D-F) mice were stained with antibodies against α-SMA (yellow) to label airway smooth muscle and myofibroblasts, PECAM1 (magenta) to label endothelial cells and DAPI to label nuclei (cyan). Sections were imaged by confocal microscopy and images are representative of samples from three independent litters. Maximum *z*-projections of anti-α-SMA labeling are shown in B,C,E and F with areas outlined by white in B and E shown at higher magnification in C and F, respectively. White arrows highlight cells with different α-SMA staining patterns. (G-J) Lung sections from PND0 WT (G,H) and *Sp3^−/−^* (I,J) mice were stained using a modified Hart's stain to visualize elastic fibers. Areas outlined by boxes in G and I are shown at higher magnification in H and J. Arrows in H and J highlight differences in fiber appearance between WT and *Sp3^−/−^* samples. Images are representative of samples from three independent litters.

To characterize the morphology of the fetal lung mesenchymal populations in WT and *Sp3^−/−^* lungs, we examined the expression pattern of α-smooth muscle actin (α-SMA) at PND0 (Fig. 6). Smooth muscle cells surrounding the conducting airways appeared to be similar in WT and *Sp3^−/−^* lungs (Fig. 6A,D). However, while the α-SMA^+^ myofibroblasts residing in the distal gas exchange regions of WT lungs contained elongated cell processes with α-SMA^+^ stress fibers (Fig. 6B,C), α-SMA^+^ cells in *Sp3^−/−^* lungs demonstrated a more rounded morphology with cytoplasmic actin staining (Fig. 6E,F). We also examined the fibrillin- and elastin-containing elastic fiber assembly in PND0 *Sp3^−/−^* lungs using a modified Hart's stain. In WT lungs, thin, long filaments of elastic fibers stretch around saccular airways (Fig. 6G,H). In contrast, elastic fibers in *Sp3^−/−^* lungs were much less abundant and present as short, wavy segments (Fig. 6I,J). Collectively, these data demonstrated the requirement of Sp3 in the formation of both the lung vasculature and mechanical, tensile support structures in the distal lung.

**Fig. 7.**
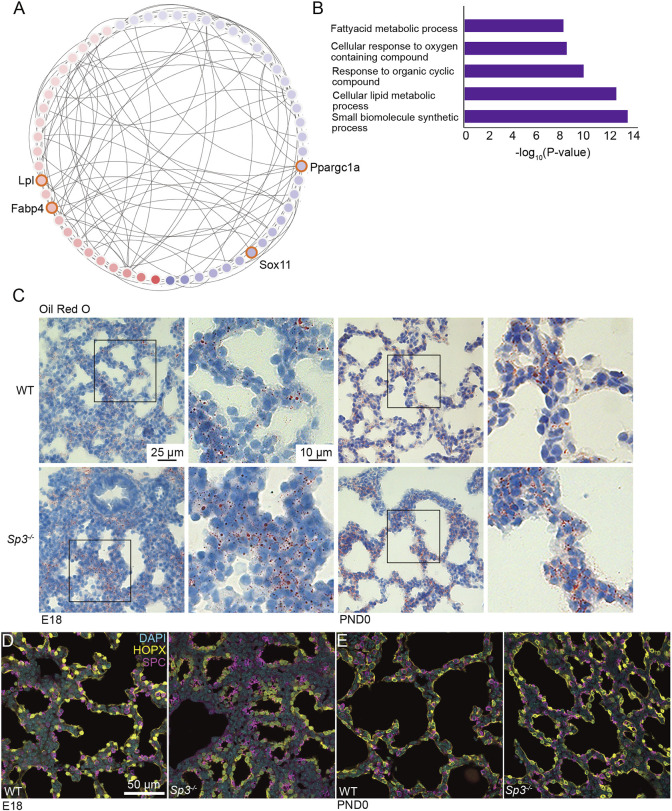
**Deletion of Sp3 increased lipid accumulation in mouse lungs.** (A) Subnetwork related to lipid and lipoprotein metabolism, with upregulated genes in *Sp3^−/−^* samples compared with WT indicated in red and downregulated genes shown in blue. (B) GO categories enriched for differentially expressed genes in this subnetwork. (C) Oil Red O staining of WT and *Sp3^−/−^* E18 and PND0 lung sections demonstrated more abundant and widespread lipid droplets throughout the lung interstitium of *Sp3^−/−^* mice. Areas outlined in boxes are shown at higher magnification in panels to the right. Images are representative of samples taken from three different litters. (D,E) Lung sections from E18 (D) and PND0 (E) WT and *Sp3^−/−^* mice were stained with antibodies against HOPX to label AT1 cells (yellow), surfactant protein C to label AT2 cells (SPC, magenta) and DAPI to label nuclei (cyan). Sections were imaged by confocal microscopy and images are representative of samples from three independent litters.

Although deletion of Sp3 reduced extracellular matrix expression, we measured increased expression of genes involved in lipid metabolism in *Sp3^−/−^* cells. Fig. 7A,B shows a subnetwork corresponding to metabolism of lipids and lipoproteins, with increased expression of the lipoprotein lipase *Lpl* and fatty acid binding protein-4 (*Fabp4*). These patterns suggested that loss of Sp3 might promote a lipofibroblast phenotype. To test this hypothesis, we stained E18 and PND0 lung sections with Oil Red O to label lipid droplets throughout lung tissue (Fig. 7C). In WT lungs, lipid droplets were detected in distinct clusters of mesenchymal cells and in epithelia, consistent with lipofibroblast localization and lamellar bodies within alveolar type 2 cells. Lungs from *Sp3^−/−^* mice had increased numbers of Oil Red O-positive droplets throughout the lung interstitium. These data were consistent with loss of Sp3 expression promoting a lipofibroblast phenotype in the developing lung mesenchyme, at the expense of myofibroblast or matrix-producing fibroblast phenotypes. FACS suggested PND0 *Sp3^−/−^* lungs had a higher percentage of CD31^+^ endothelial cells with a corresponding decrease in lineage-negative mesenchymal cells (Fig. S5). In addition, increased expression of endothelial markers *Vwf* and *Cd34* in *Sp3^−/−^* mesenchymal cells suggested Sp3 might also regulate mesenchymal-endothelial differentiation. Changes in mesenchymal cell differentiation could impact alveolar epithelial cell differentiation. We therefore stained E18 and PND0 lungs from WT and *Sp3^−/−^* mice with antibodies labeling AT1 (HOPX) and AT2 (SPC) cell populations. At E18, AT1 and AT2 cells in *Sp3^−/−^* lungs appeared to be slightly more clustered compared with WT (Fig. 7D). However, staining patterns at PND0 appeared to be similar in WT and *Sp3^−/−^* lungs (Fig. 7E). Sp3-dependent changes in lung mesenchymal cell differentiation therefore did not prevent epithelial differentiation.

### Sp3 is required for cell proliferation

**Fig. 8.**
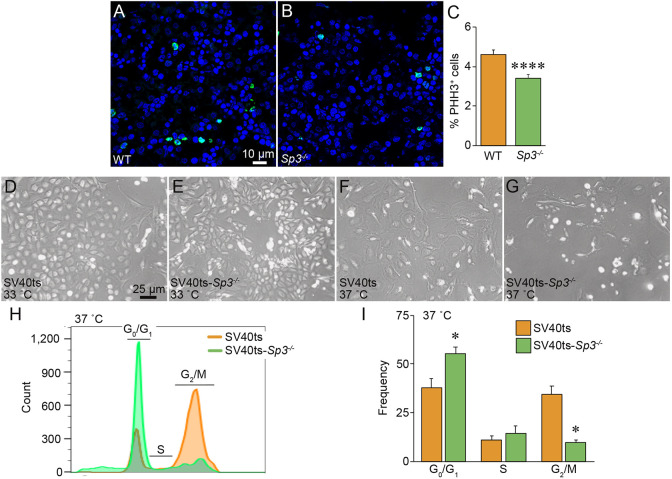
**Sp3 is required for normal cell proliferation.** (A,B) E18 WT (A) and *Sp3^−/−^* (B) liver sections were immunolabeled with an antibody against the mitotic cell marker phosphohistone H3 (PHH3, green). Nuclei were labeled with DRAQ5 (blue). Images were acquired by confocal microscopy. (C) Percentages of anti-PHH3^+^ cells were lower in *Sp3^−/−^* livers. Data are expressed as mean±s.e.m. Embryos from three separate litters were examined for each genotype, with 24 images acquired for each sample. ****P<0.0001 using two-tailed unpaired *t*-test. (D-G) Phase-contrast images of fetal lung mesenchymal cells from SV40ts and SV40ts-*Sp3^−/−^* mice cultured at 33°C (D,E) or transitioned to 37°C for 5 days (F,G). Representative images shown. (H,I) After culture at 37°C, SV40ts (orange) and SV40ts-*Sp3^−/−^* (green) cells were labeled with propidium iodide and analyzed by FACS. A representative FACS histogram is shown in H. Quantification of cell frequency within each cell cycle phase is shown in I. Data are shown as mean±s.e.m. *N*=3 independent experiments. *P<0.05 using two-tailed unpaired *t*-test.

In addition to the lung morphogenesis abnormalities, mice lacking Sp3 were smaller than their control littermates. We hypothesized this growth phenotype was due to reduced cell proliferation during embryogenesis. GSEA supported this hypothesis by identifying reduction in E2F target gene expression in *Sp3^−/−^* cells (Fig. 3). To test whether Sp3 was required for cell proliferation during development, we stained fetal tissue sections for the mitotic cell marker phospho-histone H3 (PHH3). Because very few PHH3^+^ cells were detected in fetal lung sections, we quantified proliferating cells in fetal liver samples. As seen in Fig. 8A-C, *Sp3^−/−^* mice had significantly fewer PHH3^+^ cells when normalized to total cell number, consistent with fewer cells in the fetal liver undergoing mitosis. We also observed that *Sp3^−/−^* primary cells grew more slowly in culture with limited passage capacity. Although we were able to culture sufficient cell number for the RNA-seq experiments, we used a transgenic conditional immortalization approach to better study the role of Sp3 in cell division and proliferation. We crossed Sp3 mutant mice with transgenic SV40ts mice (Immortomice) that express an interferon-inducible, temperature sensitive SV40 large T allele. The SV40ts allele allowed us to isolate primary fetal lung mesenchymal cells, expand them at 33°C in the presence of IFN-γ, and then study their ability to move through the cell cycle. Fig. 8D,E shows the similar morphology and cell density of expanding, cultured SV40ts and SV40ts-*Sp3^−/−^* cells at 33°C. Following passage, removal of IFN-γ and shift to 37°C for 5 days, SV40ts-*Sp3^−/−^* cells only partially covered tissue culture plates, suggesting a defect in cell proliferation (Fig. 8F,G). To directly study cell cycle progression, cells were then plated to reach similar densities, harvested, stained with PI, and analyzed using fluorescence-activated cell sorting (FACS). Fetal lung mesenchymal cells lacking Sp3 accumulated in G_0_/G_1_ and were less likely to advance into the G_2_/M phase (Fig. 8H,I).

**Fig. 9.**
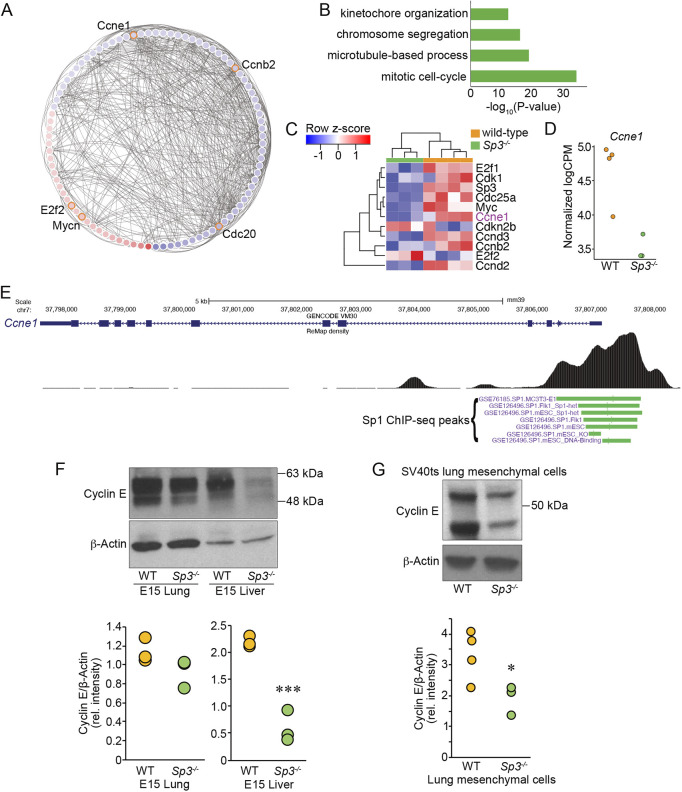
**Sp3 regulates expression of cell cycle regulators.** (A) Subnetwork with differentially expressed genes involved in cell cycle regulation. Genes upregulated in *Sp3^−/−^* cells are shown in red, genes downregulated are shown in blue. (B) GO categories of enriched differentially expressed genes in this subnetwork. (C) Heat map of known cell cycle regulators showing differential expression, with majority of transcripts downregulated in *Sp3^−/−^* cells. *Ccne1* (encoding cyclin E) is highlighted. (D) Normalized counts per million for *Ccne1* shown for each RNA-seq replicate. (E) Gene structure of the mouse *Ccne1* gene shown with Sp1 ChIP seq peaks concentrated around the promoter/enhancer region. Data from publicly available datasets via UCSC Genome Browser (mm39). (F) cyclin E immunoblot of E15 lung and E15 liver tissue homogenates from WT and *Sp3^−/−^* littermates. β-Actin shown as loading control. Densitometry of cyclin E bands (normalized to β-actin) showed lower expression in *Sp3^−/−^* liver samples compared with WT. Immunoblot is representative of three independent replicates. Data for each replicate are shown (*n*=3). ***P<0.001 using two-tailed unpaired *t*-test. (G) Cyclin E immunoblot of fetal lung mesenchymal cell lysates from SV40ts (WT) and SV40ts-*Sp3^−/−^* (*Sp3^−/−^*) mice. Cells were cultured at 37°C in the absence of IFN-γ for at least 5 days before cell lysis. β-Actin shown as loading control. Densitometry of cyclin E (normalized to β-actin) shows reduced expression in Sp3 mutant cells. Immunoblot is representative of 3-4 independent replicates. Data for each replicate are shown (*n*=4 for SV40ts, *n*=3 for SV40ts-*Sp3^−/−^*). *P<0.05 using two-tailed unpaired *t*-test.

To further investigate the molecular mechanisms linking Sp3 to alterations in cell division, we interrogated the RNA-seq dataset specifically for differentially expressed cell cycle regulators. Within the network analysis, one subnetwork was particularly enriched in cell cycle regulating transcripts (Fig. 9A,B), demonstrating the known interconnections between gene members. Fig. 9C shows the heat map of such transcripts, with *Ccne1* (encoding cyclin E, Cdk2) highlighted. The lower expression levels of *Ccne1* within each *Sp3^−/−^* sample are shown in Fig. 9D. Based on publicly available ChIP-seq datasets, the mouse *Ccne1* promoter enhancer region contains binding sites for Sp1, which would be predicted to also bind Sp3 (Fig. 9E). We also measured significantly lower levels of cyclin E protein by immunoblot in *Sp3^−/−^* fetal livers (Fig. 9F) and in cultured *Sp3^−/−^* fetal lung mesenchymal cells (Fig. 9G). While multiple genes regulating the cell cycle were differentially expressed in tissues and cells lacking *Sp3^−/−^*, the reduction in cyclin E could at least in part contribute to the observed defects in cell cycle progression and embryonal growth.

## DISCUSSION

Combining RNA-seq, *in vivo* phenotyping and cell-based studies, we identified mechanisms linking Sp3 to global gene expression, lung morphogenesis and cell proliferation. The impact of Sp3 deletion on the transcriptional program in fetal lung mesenchymal cells aligned with the developmental phenotypes observed *in vivo*. Interrogation of our RNA-seq dataset using network analysis identified diverse biological processes under the regulation of Sp3 involved in both structural lung morphogenesis and progression through the cell cycle. In the mouse model studied here, Sp3 clearly played a central role in determining cell phenotype and ultimately biological function. We propose that Sp3 helps sculpt cellular differentiation by integrating signals and inputs from growth factors, morphogens, and specific microenvironments into genome-wide transcriptional signatures.

The gas exchange regions of the developing lung require an elaborate structural support framework. Myofibroblasts and matrix fibroblasts secrete extracellular matrix proteins to provide the tensile strength within lung septa (Zhou et al., 2018; Li et al., 2020). Pericytes support the remodeling and maintenance of the alveolar capillary bed (Armulik et al., 2011; Yuan et al., 2021). Lipofibroblasts support alveolar type 2 cells within a multicellular niche (Lv et al., 2021; Taghizadeh et al., 2021; Barkauskas et al., 2013). Lungs of *Sp3^−/−^* mice had multiple findings consistent with loss of normal mesenchymal structure and cell identity. *Sp3^−/−^* lungs lacked normal myofibroblast morphology and elastic fiber formation. The changes in lung structure appeared to result from Sp3-dependent loss of normal lung mesenchymal cell differentiation and function. Sp3 deletion reduced expression of multiple extracellular matrix components. Many of these transcripts, including *Fn1*, *Tnc* and various Col family members play key roles in establishing airway branching, vascular patterning and alveolar septation during lung morphogenesis (Gremlich et al., 2020; Mund and Schittny, 2020; Sakai et al., 2003; Plosa et al., 2014). Lung mesenchymal cells expressing and releasing extracellular matrix proteins could contain activated lung myofibroblasts or a distinct subpopulation of ‘matrix’ fibroblasts (Endale et al., 2017; Green et al., 2016). Although expression of matrix components was consistently lower in *Sp3^−/−^* mesenchymal cells, markers of myofibroblast differentiation were more mixed. Cells lacking Sp3 expressed lower levels of *Acta2* and *Cxcl12*, but higher levels of *Serping1* and *Timp3*. Confocal imaging also demonstrated that PECAM1^+^ endothelia lacked the normal long interconnections throughout the saccular stage mesenchyme. The vascular defect could be due to endothelial and/or mesenchymal phenotypes. We have previously demonstrated that fetal lung mesenchymal cells possess endothelial differentiation potential, but we have yet to determine the potential role of Sp3.

Among the external factors regulating lung mesenchymal cell phenotypes, TGFβ can promote myofibroblast differentiation and contribute to pathological lung fibrosis (Hewlett et al., 2018). Downstream of TGFβ signaling, activated Smad family members cooperate with Sp1 to drive gene transcription, representing a potential mechanistic target for Sp3 (Moustakas et al., 2002). Although total lung measurements did not detect a difference in the expression of TGFβ ligands or receptors, changes in specific cell populations could still be present. In lung mesenchymal cells, *Tgfb2* and the accessory TGFβ receptor *Eng* were both lower in *Sp3^−/−^* samples, suggesting potential Sp3-dependent mechanisms that could regulate mesenchymal phenotype.

Within the mesenchyme, lipofibroblasts play key functions in lung development and homeostasis. Lipid accumulated and released by lipofibroblasts supports surfactant production by AT2 cells (Lv et al., 2021). In the adult lung, lipofibroblasts can also promote proliferation of immediately adjacent AT2 cells (Barkauskas et al., 2013). In WT lungs, lipid accumulation was seen in select interstitial regions consistent with the presence of lipofibroblasts. However, in *Sp3^−/−^* lungs, lipid droplets were more prominent and widespread throughout the lung mesenchyme. Transcriptional profiling also identified increased lipofibroblast-associated gene expression in *Sp3^−/−^* cells. We therefore propose a mechanism for Sp3 in guiding mesenchymal cell phenotype along the myofibroblast-lipofibroblast axis. Sp3 could work to integrate signals known to drive this differentiation process. Evidence supports the roles of Sp1 and Sp3 in regulating cellular differentiation in other tissues. During hematopoiesis, Sp1 and Sp3 are thought to stabilize overall promoter structures and allow efficient differentiation of precursor cells into terminally differentiated cells (Gilmour et al., 2019).

Although we measured changes in multiple Wnt ligands and increased *Axin2* expression in primary *Sp3^−/−^* mesenchymal cells, these differences were not detected in total lung samples. The significance of potential changes in Wnt signaling will require higher resolution testing *in vivo* where multicellular niches and tissue microenvironments are preserved. As GC boxes are present within a high percentage of promoter and enhancer sequences, we purposefully used in depth sequencing of RNA-seq libraries to measure transcription across the entire genome. Although this approach generated a robust dataset and identified many genes impacted by Sp3 deletion, we still need to incorporate cell-cell interaction variables to fully understand how Sp3 regulates tissue morphogenesis. Even brief periods of culture can change transcriptional signatures (Honda et al., 2022; Gosselin et al., 2017), justifying more in-depth analysis of *in vivo* transcription within specific cell populations.

Loss of Sp3 led to decreased expression of multiple cell cycle regulators, including *E2f1*, *Cdk1*, *Cdc25a*, *Myc*, *Ccne1*, *Ccnb2*, *Ccnd2* and *Ccnd3*. Reduced expression of these genes could explain the accumulation of *Sp3^−/−^* cells in G_0_/G_1_, reduced PHH3 staining in *Sp3^−/−^* tissue and small size of *Sp3^−/−^* embryos. The impact is clearly not absolute, as *Sp3^−/−^* embryos do form and survive until delivery, unlike *Sp1^−/−^* embryos which fail to develop past E10 (Marin et al., 1997). Our data presented here give important new insight into the molecular mechanisms linking cell proliferation and Sp3 function. Numerous studies have measured increased Sp family member expression and activity in cancer cells and tissues (reviewed by Vizcaino et al., 2015). Inhibition of both Sp1 and Sp3 DNA binding with mithramycin slows cell proliferation in multiple cell models (Huang et al., 2020; Quarni et al., 2019; Liu et al., 2018). As Sp1 and Sp3 are known to bind the same DNA motifs within promoter and enhancer regions (Kingsley and Winoto, 1992), their different roles in regulating embryo growth may involve association with other distinct transcriptional regulators.

Unlike Sp1, which largely activates gene transcription via GC box binding, Sp3 can either activate or inhibit gene expression (Hagen et al., 1994; Majello et al., 1997; Birnbaum et al., 1995). The mechanisms guiding the inhibitory role of Sp3 are unclear. Our RNA-seq data illustrate the repressive role of Sp3, with higher expression of many transcripts in *Sp3^−/−^* cells. Activation and nuclear transport of NF-κB activation led to decreased *Fgf10* expression via interactions with Sp3 and not via a discrete NF-κB binding site (Carver et al., 2013). This inhibitory effect required sequences in the N-terminal region of Sp3, away from the conserved C-terminal DNA binding region. In Alzheimer's disease tissues and models, the interactions between Sp3 and HDAC2 regulate synaptic plasticity via repression of multiple transcripts involved in synapse formation (Yamakawa et al., 2017). We speculate that Sp3 acts to provide inhibitory factors access to gene promoters and enhancer regions. The unique regions in the Sp3 N-terminus likely instruct this repressive activity, whereas the conserved DNA-binding zinc finger region determines GC box specificity similar to Sp1, which functions primarily as an activator of transcription. Our data implicate Sp3 as a key transcriptional regulator of cellular differentiation and proliferation during development.

## MATERIALS AND METHODS

### Reagents

The following reagents were used for immunofluorescence: rat anti-platelet endothelial cell adhesion molecule 1 (PECAM1; BD Pharmingen, 553370, 1:250-1000), mouse anti-α-smooth muscle actin-Cy3 (Sigma-Aldrich, C6198, 1:100-1000), rat anti-E-cadherin (Invitrogen, 53-3249-82, 1:500), rabbit anti-phosphohistone H3 (EMD Millipore, 06-570, 1:250) mouse anti-HOPX (E-1; Santa Cruz Biotechnology, sc-398703, 1:100), rabbit anti-SPC (Abcam, ab90716, 1:500). Prolong Gold mounting media (±DAPI) and Alexa Fluor fluorescent secondary antibodies (Alexa 488 donkey anti-rat A21208, Alexa 594 donkey anti-rabbit A21207, both diluted to 1:1000) were purchased from Invitrogen. The nuclear stain DRAQ5 (62251, 1:5000) was purchased from Thermo Fisher Scientific. Antibody titrations were performed on WT tissues to determine the optimal dilution for each tissue. Cells and explants were cultured in the following reagents: interferon-γ (R&D Systems, 485-MI-100), Dulbecco's modified Eagle's medium (DMEM; Corning Life Sciences, 10-013-CV), fetal bovine serum (FBS; Thermo Fisher Scientific), and penicillin-streptomycin (Thermo Fisher Scientific, 15140122).

### Mice

All experiments were approved by the Institutional Animal Care and Use Committees at the University of California, San Diego and Stanford University. Sp3 heterozygous mice were kindly provided by Professor J.N.J. Philipsen (Erasmus University Medical Center, Rotterdam, Netherlands) and mice expressing the temperature-sensitive early region SV40 mutant tsA58 allele (SV40ts; Immortomice) were purchased from Charles River Laboratories. Genotyping was performed by standard PCR. For timed matings, E0 was identified as the morning of vaginal plug confirmation. PND0 was defined as the day of delivery. Live video monitoring was used to ensure PND0 mice were collected immediately after birth.

### Cell and explant culture

For isolation of fetal lung mesenchymal cells, fetal lungs were dissected and enzymatically digested with collagenase (0.7 mg/ml). Cells were passed through a 70 µm strainer and pelleted by centrifugation at 400 ***g***. Mesenchymal populations were isolated by rapid adhesion to tissue culture plastic and subsequent passage. Isolated fetal lung mesenchymal cells from SV40ts mice were maintained at 33°C in DMEM with 10% FBS with penicillin/streptomycin supplemented with IFN-γ. All cells were moved to 37°C and passaged at least once before experiments. Fetal lung explants were isolated and cultured as previously described (Stouch et al., 2016; Prince et al., 2005). Brightfield images were captured at 24 and 72 h of culture. Branch count analysis was performed using ImageJ software version 1.51 (National Institutes of Health).

### Tissue processing and immunostaining

Fetal and newborn mouse lungs were dissected, fixed in 4% paraformaldehyde (Electron Microscopy Sciences) and paraffin embedded. Sections were then stained with hematoxylin (Dako North America), eosin (Ricca Chemical), modified Hart's stain (reagents from Electron Microscopy Sciences) or Oil Red O (IHC World). Volumetric measurement of lung tissue and airspace volume of hematoxylin and eosin-stained section was performed using ImageJ. For immunostaining, fetal mouse lungs were fixed in 4% paraformaldehyde, washed and processed through a sucrose gradient before being embedded in OCT freezing media (Tissue-Tek; Sakura Finetek USA). Frozen sections were stained with antibodies of interest followed by Alexa-conjugated secondary antibodies, and nuclei were stained with DRAQ5.

### Imaging and image analysis

Confocal images were acquired using a Leica TCS SPE laser scanning confocal microscope. Brightfield images were obtained using Olympus IX81, Leica DMiL or Leica DMi8 inverted microscopes with color CCD cameras. All images were saved and imported to ImageJ or Photoshop software version CS6 (Adobe Systems) for processing. Identical image processing parameters were used for achieving proper image comparison.

### RNA isolation and RNA-seq

RNA was isolated using TRIzol (Ambion; Life Technologies) reagent and quantified with a Nanodrop 1000 (Thermo Fisher Scientific). RNA quality was determined using an Agilent 4200 TapeStation (Agilent Technologies). Samples with high RIN scores (>7) were selected for preparation of libraries with TruSeq RNA Library Prep Kit (Illumina) using 10 ng of RNA. Libraries were sequenced with 50 bp single end reads to a depth of 30 million reads per sample. Analysis was performed by the University of California, San Diego (UCSD) Center for Computational Biology & Bioinformatics.

### Lung RNA expression analysis

Total RNA was isolated from snap frozen lung tissue using TRIzol. RNA was converted into cDNA using SuperScript III First-Strand Synthesis System (Invitrogen). Quantitative PCR was performed using predesigned primers (Table S1; IDT and Bio-Rad) and iQ SYBR Green Supermix (Bio-Rad) on a QuantStudio 6 Pro Real-Time PCR System (Applied Biosystems). Gene expression levels were represented using the 2^−ΔCT^ method and normalized to *Gapdh*. When a target gene was undetectable for a sample, it was given a C_T_ value of 40 for that gene for statistical comparison and plotting purposes.

### Bioinformatic analysis and data availability

#### RNA-seq data analysis

Quality control of the raw fastq files was performed using the software tool FastQC v0.10.1 (https://www.bioinformatics.babraham.ac.uk/projects/fastqc/). Sequencing reads were aligned to the mouse genome (mm10) using the STAR v2.4.5a aligner (Dobin et al., 2013). Read quantification was performed with htseq-count. The R BioConductor packages edgeR (Robinson et al., 2010) and limma (Ritchie et al., 2015) were used to implement the limma-voom (Law et al., 2014) method for differential expression analysis. Lowly expressed genes were filtered out [counts per million (CPM)>1 in at least one sample]. Trimmed mean of M-values (TMM; Robinson and Oshlack, 2010) normalization was applied. The experimental design was modeled upon time point and treatment (∼0 +genotype_condition). The lmFit function in limma with consensus correlation to account for repeated measures of patient followed by the eBayes function was used to fit the design on voom normalized counts per gene. Significance was defined by using an adjusted *P*-value cut-off of 0.05 after multiple testing correction using a moderated *t*-statistic in Limma. GSEA was conducted with WebGestalt (Liao et al., 2019; Subramanian et al., 2005). RNA-seq data have been deposited at the Gene Expression Omnibus (GSE225703).

#### Network analysis

Differentially expressed genes between *Sp3^−/−^* and WT cells (defined as those with an adjusted *P*-value<0.05 and an absolute log2-Fold Change>1) were input into Mouse StringDB v11.0 (Szklarczyk et al., 2019) to extract the high confidence interaction network among these genes. We applied Louvain clustering (Blondel et al., 2008) to identify subnetworks of closely interconnected genes and visualized this with Cytoscape (Shannon et al., 2003). The network was uploaded to NDex (https://ndexbio.org/viewer/networks/54aeb0f1-eb39-11eb-b666-0ac135e8bacf) (Pratt et al., 2015). Enrichment analysis of each cluster was performed with WebGestalt.

#### Cell cycle progression and apoptosis

SV40ts fetal lung mesenchymal cells were cultured for 5 days at 37°C in the absence of IFN-γ. Cells were trypsinized and fixed in ethanol overnight. After fixation, cells were stained with propidium iodide solution (Cell Signaling Technology) and analyzed using the BD FACSCanto II. Data analysis was performed using FACSDiva and FlowJo software.

#### FACS of lung cell populations

Following euthanasia, newborn (PND0) mouse lungs were perfused intracardially through the right ventricle with cold PBS. Lung tissue was minced and enzymatically digested in RPMI-1640 (Corning 10-040-CV) containing 50 U/ml DNase I (Roche, 10104159001), 400 µg/ml Liberase TM (Roche, 5401119001) at 37°C for 15 min. Tissue homogenates were passed through a 100 µm cell strainer (Thermo Fisher Scientific), followed by erythrocyte lysis using ACK buffer (Life Technologies). Before antibody staining, the cell suspensions were filtered through a 70 µm cell strainer (Thermo Fisher Scientific). Single cell suspensions were resuspended in PBS with CD16/CD32 blocking antibody (BioLegend, 101320) and Zombie Aqua (BioLegend, 423102) for 20 min. The cells were then incubated with fluorochrome-conjugated antibodies against CD45, CD31 and EPCAM (BioLegend, 109828, 102507 and 118215, respectively) in staining buffer (HBSS containing 2% FBS and 2 mM EDTA) for 1 h. Samples were analyzed on a NovoCyte Penteon Flow Cytometer (Agilent). Data were analyzed using FlowJo software.

#### Immunoblotting

Whole lung and liver tissue was snap-frozen in liquid nitrogen following dissection and stored at −80°C. Samples were thawed on ice in RIPA buffer (Thermo Fisher Scientific) containing protease inhibitors (Halt Protease Inhibitor Cocktail, 87785, Thermo Fisher Scientific) and homogenized using a mechanical tissue disruptor. Fetal lung mesenchymal cells were washed and collected in ice-cold RIPA buffer with protease inhibitors. Samples were centrifuged and protein lysate concentrations were quantified using BCA Assay Kit (Thermo Fisher Scientific). Lysates were separated on SDS polyacrylamide gels and transferred onto polyvinylidene difluoride (PVDF) membranes (Bio-Rad). Membranes were first blocked with 5% bovine serum albumin (Thermo Fisher Scientific) in 0.1% Tween 20 (Thermo Fisher Scientific) for 1 h at room temperature. Following blocking, membranes were incubated overnight at 4°C in the blocking buffer with antibodies against cyclin E (Santa Cruz Biotechnology) and β-actin (Sigma-Aldrich). After primary antibody incubation, membranes were first washed with Tris-buffered saline containing 0.1% Tween 20 and then incubated with HRP-conjugated secondary antibodies for 1 h at room temperature. Immunoblots were visualized by standard ECL (Thermo Fisher Scientific). Developed autoradiographs were then scanned for densitometry.

## Supplementary Material

10.1242/develop.200839_sup1 Supplementary information
