# Supplementary material for: Sp3 is essential for normal lung morphogenesis and cell cycle progression during mouse embryonic development
Source: Development. 2023 Mar 3;150(5):dev200839. doi: 10.1242/dev.200839 (PMC10110423; doi:10.1242/dev.200839)
Supplement: Supplementary information [file develop-150-200839-s1.pdf]

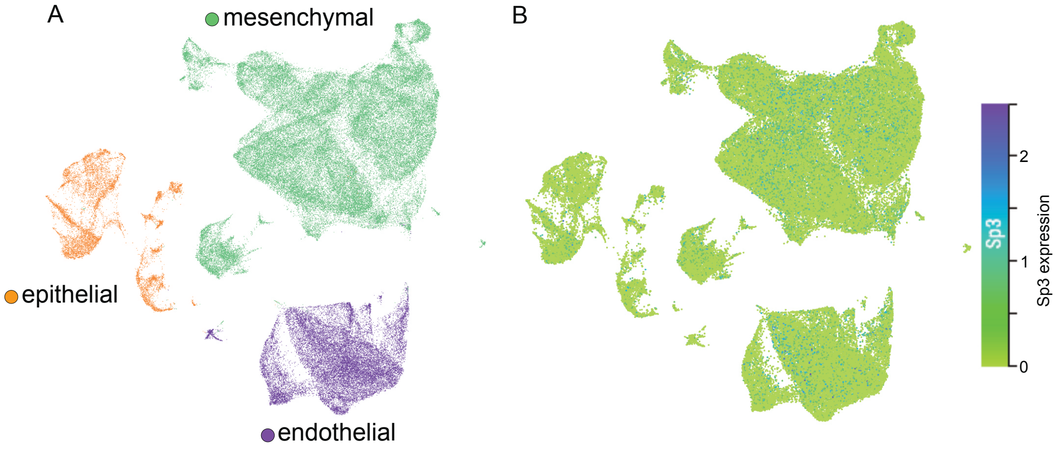

Fig. S1. A. UMAP plot of fetal mouse lung single cell RNAseq data originally reported (Negretti et al., 2021) and now publicly available through GEO. **B.** Sp3 expression throughout all lung cell populations. https://lungcells.app.vumc.org/

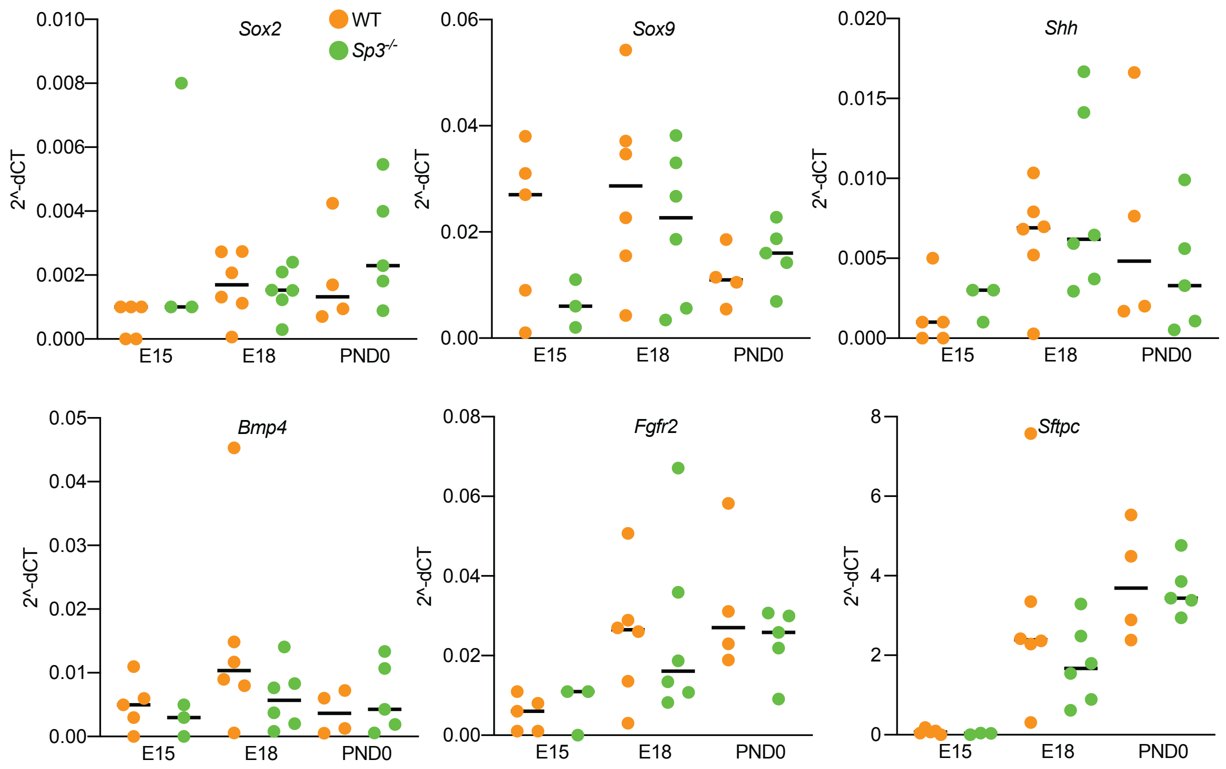

Fig. S2. Real time PCR data comparing epithelial marker gene expression in WT and *Sp3*$^{-/-}$ fetal (E15 and E18) and newborn (PND0) mouse lung tissue. Total RNA was isolated from lung homogenates and gene expression measured using commercially validated primer sets. Data for each independent biological replicate shown. Gene expression levels plotted as $2^{-\Delta CT}$ after normalization of C_T values to *Gapdh* for each replicate.

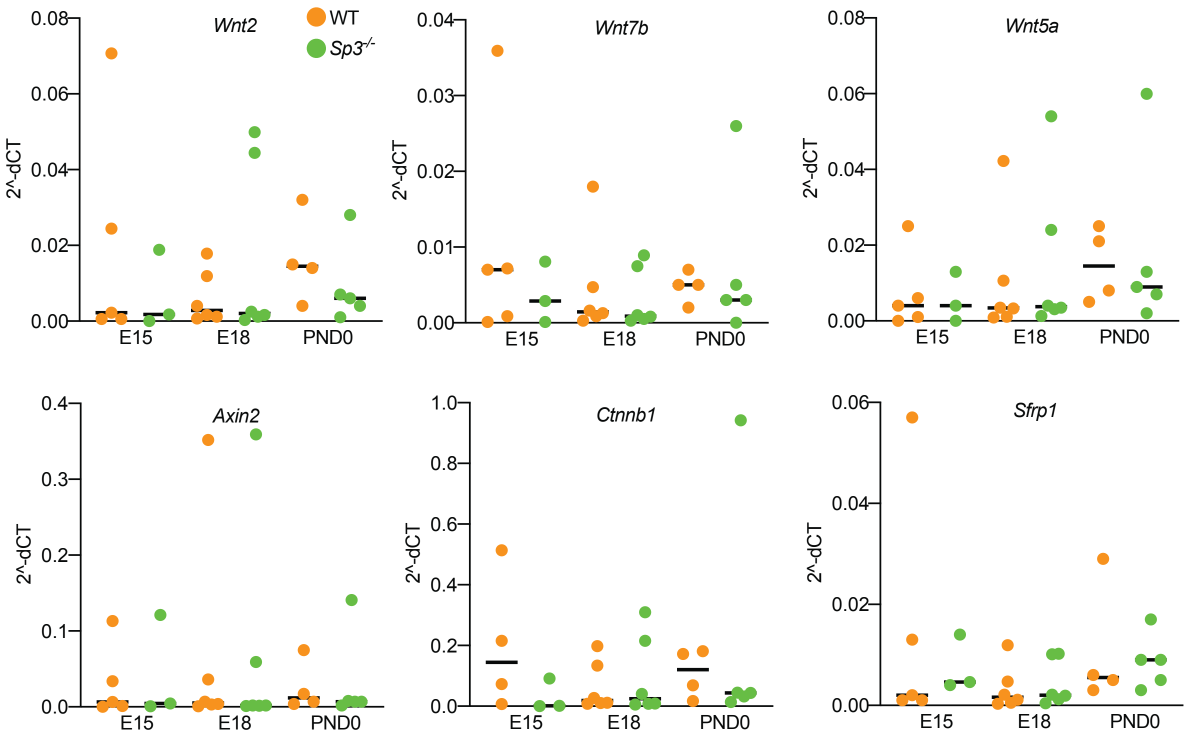

Fig. S3. Real time PCR data comparing Wnt signaling gene expression in WT and *Sp3*$^{-/-}$ fetal (E15 and E18) and newborn (PND0) mouse lung tissue. Total RNA was isolated from lung homogenates and gene expression measured using commercially validated primer sets. Data for each independent biological replicate shown. Gene expression levels plotted as $2^{-\Delta CT}$ after normalization of C_T values to *Gapdh* for each replicate.

Development • Supplementary information

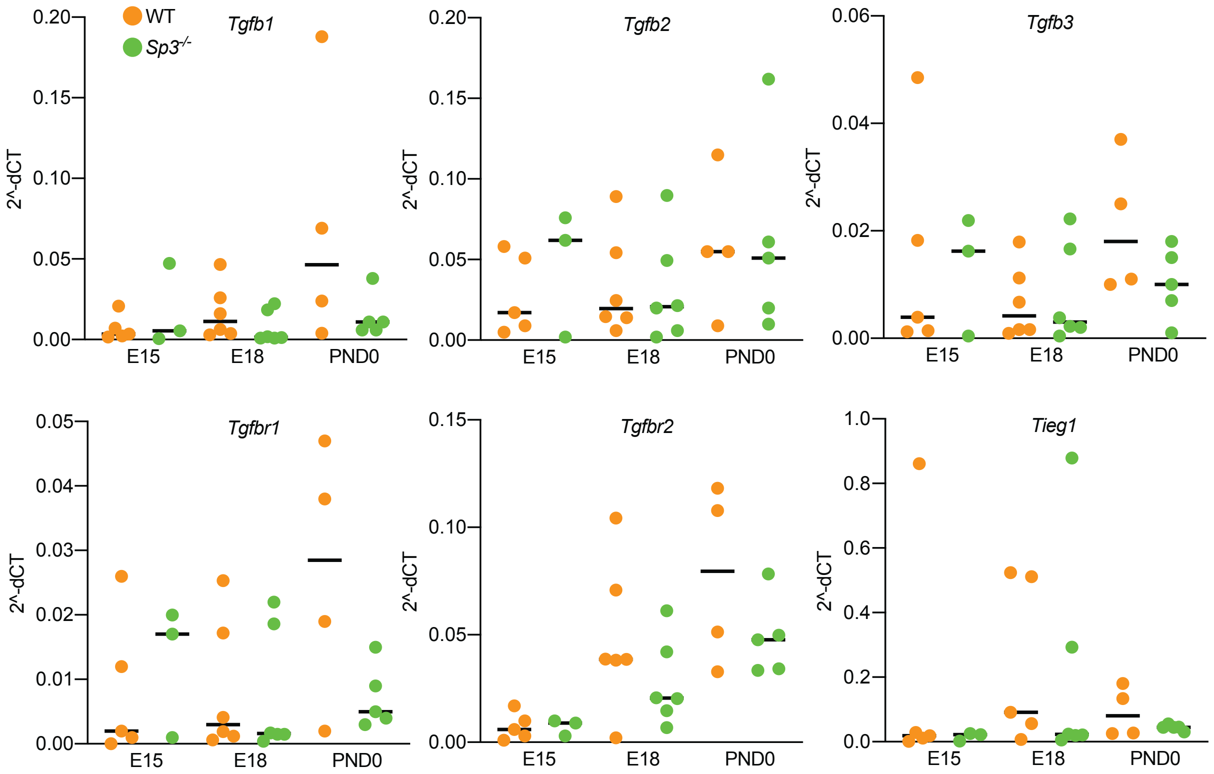

Fig. S4. Real time PCR data comparing TGFβ signaling gene expression in WT and *Sp3*$^{-/-}$ fetal (E15 and E18) and newborn (PND0) mouse lung tissue. Total RNA was isolated from lung homogenates and gene expression measured using commercially validated primer sets. Data for each independent biological replicate shown. Gene expression levels plotted as $2^{-\Delta CT}$ after normalization of C_T values to *Gapdh* for each replicate.

Development • Supplementary information

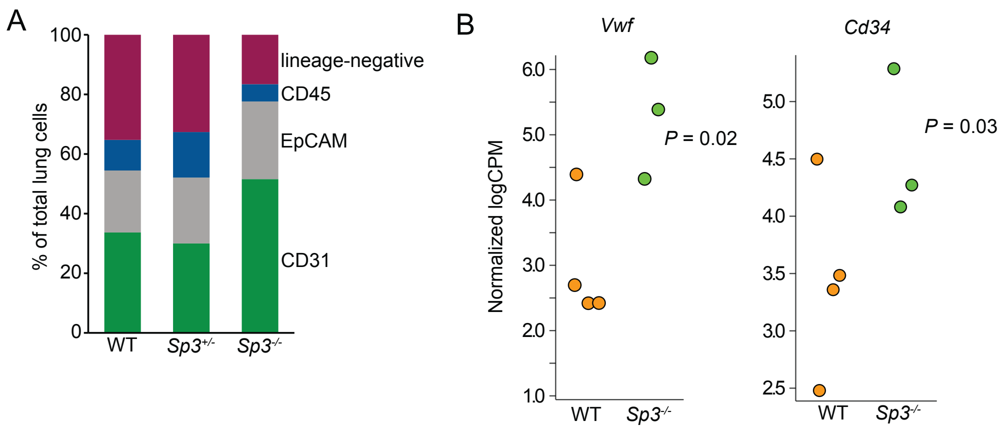

Fig. S5. A. FACS of total lung cell populations from PND0 mouse lungs. Single cell suspensions from WT, *Sp3*$^{+/-}$, and *Sp3*$^{-/-}$ lungs were labeled with antibodies against CD45 (immune cells), EpCAM (epithelial cells), and CD31 (endothelial cells). Lineage negative cells contained various mesenchymal cells. **B.** RNAseq data from fetal lung mesenchymal cells showing expression of the endothelial markers *Vwf* and *Cd34* in WT and *Sp3*$^{-/-}$ mesenchymal cells.

Development • Supplementary information

Table S1. qPCR primers

Gene	Company	Catalog #
Axin2	IDT	421924103
Bmp4	IDT	421924082
Ctnnb1	IDT	421924106
Etv4	IDT	421924076
Etv5	IDT	421924070
Fgf10	IDT	421924073
Fgfr2	BioRad	qMmuCED0047697
Sfrp1	IDT	421924109
Sftpc	IDT	421924091
Shh	IDT	421924079
Sox2	IDT	421924085
Sox9	IDT	421924088
Tbx4	IDT	421924112
Tgfb1	IDT	421924115
Tgfb2	IDT	421924118
Tgfb3	IDT	421924121
Tgfbr1	IDT	421924124
Tgfbr2	IDT	421924127
Wnt2	IDT	421924094
Wnt5a	IDT	421924097
Wnt7b	IDT	421924100

Supplemental Material References

NEGRETTI, N. M., PLOSA, E. J., BENJAMIN, J. T., SCHULER, B. A., HABERMANN, A. C., JETTER, C. S., GULLEMAN, P., BUNN, C., HACKETT, A. N., RANSOM, M., TAYLOR, C. J., NICHOLS, D., MATLOCK, B. K., GUTTENTAG, S. H., BLACKWELL, T. S., BANOVICH, N. E., KROPSKI, J. A. & SUCRE, J. M. S. 2021. A single-cell atlas of mouse lung development. *Development,* 148.

Development • Supplementary information
