# Supplementary material for: Sp3 is essential for normal lung morphogenesis and cell cycle progression during mouse embryonic development
Source: Development. 2023 Mar 3;150(5):dev200839. doi: 10.1242/dev.200839 (PMC10110423; doi:10.1242/dev.200839)
Supplement: Supplementary file 1 [file develop-150-200839_review_history.pdf]

Sp3 is essential for normal lung morphogenesis and cell cycle progression during mouse embryonic development

Alyssa M. McCoy, Omar Lakhdari, Sayane Shome, Kaitlin Caoili, Gilberto E. Hernandez, Nima Aghaeepour, Lindsay D. Butcher, Kathleen Fisch and Lawrence S. Prince

DOI: 10.1242/dev.200839

Editor: Liz Robertson

Review timeline

Original submission:	8 April 2022
Editorial decision:	17 May 2022
First revision received:	5 January 2023
Accepted:	18 January 2023

Original submission

First decision letter

MS ID#: DEVELOP/2022/200839

MS TITLE: Sp3 Is Essential for Normal Lung Morphogenesis and Cell Cycle Progression During Mouse Embryonic Development

AUTHORS: Alyssa M. McCoy, Omar Lakhdari, Sayane Shome, Kaitlin Caoili, Gilberto E E. Hernandez, Nima Aghaeepour, Kathleen Fisch, and Lawrence S. Prince

I have now received all the referees' reports on the above manuscript, and have reached a decision. The referees' comments are appended below, or you can access them online: please go to BenchPress and click on the 'Manuscripts with Decisions' queue in the Author Area.

As you will see, the referees express considerable interest in your work, but have some significant criticisms and recommend a substantial revision of your manuscript before we can consider publication. If you are able to revise the manuscript along the lines suggested, which may involve further experiments, I will be happy receive a revised version of the manuscript. Your revised paper will be re-reviewed by one or more of the original referees, and acceptance of your manuscript will depend on your addressing satisfactorily the reviewers' major concerns. Please also note that Development will normally permit only one round of major revision. If it would be helpful, you are welcome to contact us to discuss your revision in greater detail. Please send us a point-by-point response indicating your plans for addressing the referee's comments, and we will look over this and provide further guidance.

Please attend to all of the reviewers' comments and ensure that you clearly highlight all changes made in the revised manuscript. Please avoid using 'Tracked changes' in Word files as these are lost in PDF conversion. I should be grateful if you would also provide a point-by-point response detailing how you have dealt with the points raised by the reviewers in the 'Response to Reviewers' box. If you do not agree with any of their criticisms or suggestions please explain clearly why this is so.

Reviewer 1

Advance summary and potential significance to field

© 2023. Published by The Company of Biologists under the terms of the Creative Commons Attribution License (https://creativecommons.org/licenses/by/4.0/).

The paper by Alyssa M McCoy et al. reports the role of Sp3 during lung development. Using a global KO approach, they further analyzed the lung phenotype of Sp3-/- vs. Sp3+/+ (WT) embryos at E15, E18 and immediately after birth. They used bulk RNAseq to tease out the transcriptomic changes in the lung mesenchyme at E15. They also provide evidence for impaired lung vasculature, decreased mesenchymal proliferation, abnormal myofibroblast differentiation and increased lipofibroblast formation.

Comments for the author

The paper by Alyssa M McCoy et al. reports the role of Sp3 during lung development. Using a global KO approach, they further analyzed the lung phenotype of Sp3-/- vs. Sp3+/+ (WT) embryos at E15, E18 and immediately after birth. They used bulk RNAseq to tease out the transcriptomic changes in the lung mesenchyme at E15. They also provide evidence for impaired lung vasculature, decreased mesenchymal proliferation, abnormal myofibroblast differentiation and increased lipofibroblast formation.

In general, the paper is well written and the results are convincing.

Major critiques

1_ It would be informative to quantify the expression of Sp3 in the epithelium and mesenchyme during lung development over time to give some context to the work. Is Sp3 expressed at higher level in the mesenchyme? In which mesenchymal clusters? The use of available data set (scRNAseq or bulk RNAseq) should render this feasible and cost effective.

2_ Figure 1: the comparison of the branching process at E15 between WT and KO clearly indicates a branching defect already. The authors should quantify this. In addition, it may be informative to look at earlier time points (E12.5) to see if the defects are already present. This would give the authors the opportunity to look for the classical genes involved in the branching process (Fgf10, Fgfr2b, Etv4/5, Shh, etc...). Is Sp3 involved in the control of these canonical genes regulating the branching process? The heatmap in figure 4B (looking at developmental signaling is very informative for the branching process as it shows reduced Fgf10 expression at E15.5. Does this translate into less proliferation in the lung epithelium at E15.5? The authors should also look at markers of epithelial differentiation (proximal and distal markers of differentiation). This would make their paper very informative. Is the epithelium just paying the price of mesenchymal defects due to Sp3 deletion in the mesenchyme or are they cell-autonomous defects in the epithelium?

3_ What is the significance of increased Axin2 expression in Sp3-/- mesenchyme? Is more Wnt signaling (which needs to be demonstrated) consistent with the phenotypes described in terms of proliferation and differentiation?

4_ Figure 3B: for the heatmap, the authors should first show the Wild type and then the Sp3ko samples (like for the heatmaps above and underneath).

5_ The increase in Lipofibroblast number is Sp3ko lungs is very puzzling as the authors show also a decrease in Fgf10 expression (Figure 4B). Could this be due to decreased TGFb signaling (Tgfb signaling has also been proposed to prevent LIF formation). It would also be interesting to show if the mesenchyme from Sp3Ko lungs could be more supportive for organoid formation (use of postnatal AT2s may be possible here but would need to be established). A general question from this reviewer is: what are the consequences of increased LIF on the AT2s?

6_ It is not clear why the authors do not show directly proliferation in the lung mesenchyme in situ (Figure 8 A-C: they show instead the liver). An explanation should at least be given for this choice. Why did the authors feel the need to immortalize the lung mesenchyme cells?

7_ Unless I missed it, it seems that the bulk RNAseq analysis was done only at E15.5, it would be interesting to do it also at E18.5 and P0. If it is possible to generate more KO embryos, it would be great to quantify the defect in the epithelium and mesenchyme by FACS to quantify the abundance of different subpopulations.

© 2023. Published by The Company of Biologists under the terms of the Creative Commons Attribution License (https://creativecommons.org/licenses/by/4.0/).

Reviewer 2

Advance summary and potential significance to field

In this manuscript, McCoy et al. used Sp3-/- mouse embryos and cultured lung mesenchymal cells to demonstrate that SP3 transcription factor is essential for normal lung morphogenesis and cell cycle progression during mouse embryonic development. RNAseq and cellular phenotyping in Sp3-/- embryos identified changes in extracellular matrix deposition, cell proliferation, and fibroblast differentiation which resulted in multiple structural defects, including lung vascular abnormalities, lack of elastic fiber formation and accumulation of lipids within lung fibroblasts. Based on in vitro and in vivo data, Sp3-/- cells exhibited cell cycle arrest with accumulation of cells in G0/G1 phase of the cell cycle, which was due to decreased expression of multiple cell cycle regulators, including Cyclin E, implicating SP3 in activation of Cyclin E expression. Overall, the present study convincingly demonstrates the global impact of SP3 on lung embryonic development in the mouse. The manuscript contains novel and high-quality experimental data, and main conclusions are supported by experimental data, identifying SP3 as a novel regulator of lung development.

Comments for the author

Several points need to be addressed to improve overall quality if the manuscript:

Specific points:

1. It is unclear what is the rationale for brief culturing of lung mesenchymal cells prior to RNAseq. Is this for purification purposes? The authors should consider and discuss a possibility that cell culture conditions can change gene expression in lung mesenchyme.

2. Vascular changes and the capillary density in E15 lungs should be quantitated, preferably, in 3D.

3. In Fig. 5D, the authors refer to “elongated cellular processes”. This should be corrected. Low magnification images show only the location of endothelial cells and basic structure of the capillary network. The endothelial cell processes cannot be clearly seen at these low magnification images. Additional high magnification images are needed in Fig. 5.

4. Western blot in Fig. 9E clearly shows a decrease in Cyclin E protein in E15 Sp3-/-lungs. However quantification of these data does not indicate statistically significant differences in E15 Sp3-/- lungs because the blot looks over-exposed. This leads to overall impression that the quantification is not consistent with Western blot images.

5. It is unclear whether SP3 regulates Cyclin E expression through direct (transcriptional) or indirect mechanisms.

Reviewer 3

Advance summary and potential significance to field

This manuscript by McCoy et al. describes the effect of deleting Sp3 on mouse lung development. the authors show that deletion of Sp3 impairs vascular and mesenchymal development in the mouse lung. Imbalance in myofibroblast/lipofibrobalsts differentiation as well as defects in vascular development are seen following Sp3-/-. Detailed transcriptional profiling and thorough analyses of Sp3-/- fetal lung mesenchymal cells provides insight into the genes regulated by Sp3.

Comments for the author

This manuscript by McCoy et al. describes the effect of deleting Sp3 on mouse lung development. While the study is very descriptive, the experiments were carefully carried out and the findings include detailed transcriptional profiling and validation of genes and pathways altered by the absence of Sp3. While the study is interesting, several issues need to be addressed by the authors.

© 2023. Published by The Company of Biologists under the terms of the Creative Commons Attribution License (https://creativecommons.org/licenses/by/4.0/).

Major:

Was there a difference in lung size at E15?
Since the authors show branching defects in cultured explants, it would be interesting to know at what stage the lung defects start? And whether there is overall less branches at E12 and/or E14?

Authors claim that Sp3-/- explants “displayed thicker interstitial regions”. H7E to support this claim should be provided in Figure 1.

Authors should provide additional details on passage number for the mesenchymal cells that were transcriptionally profiled. What is “brief culture period”? were these cells treated with IFN-g in culture?

Page 9, line 174. It would be helpful if the authors cited in the results section examples of those differentially expressed genes that are markers of lipo- and myo- fibroblasts phenotypes. Alternatively, a more detailed description of results and data in Figure 4 could be provided.

The authors should include either an excel sheet detailing all the differentially expressed genes or make their sequencing data available online.

Figure 6: The claim of rounder a-SMA+ cells isn’t convincing based on the figures shown. In addition, what is the difference between panels B and C and panels E and F?

The rationale for staining liver sections for cell proliferation in Figure 8 isn’t clear. The authors have lung sections available, why did they not stain lung sections?

Minor:

Please include Scale bars for Figure 1D

Figure 5 B and D, the nuclear staining should be improved. Scale bar size should be verified. Panel D should be replaced with a similar region/area as to what is shown in panel A

Figure 6 panel J: please provide a higher resolution image

First revision

Author response to reviewers' comments

We appreciate the overall enthusiasm for our manuscript and are thankful for the detailed review. The comments from the reviewers are insightful and our revised manuscript includes additional experimental data and significant changes to the text that we feel address each of the questions and comments. Below we have included the entire review text along with our specific responses.

Reviewer 1 Advance Summary and Potential Significance to Field:

The paper by Alyssa M McCoy et al. reports the role of Sp3 during lung development. Using a global KO approach, they further analyzed the lung phenotype of Sp3-/- vs. Sp3+/+ (WT) embryos at E15, E18 and immediately after birth. They used bulk RNAseq to tease out the transcriptomic changes in the lung mesenchyme at E15. They also provide evidence for impaired lung vasculature, decreased mesenchymal proliferation, abnormal myofibroblast differentiation and increased lipofibroblast formation.

Reviewer 1 Comments for the Author:

The paper by Alyssa M McCoy et al. reports the role of Sp3 during lung development. Using a global KO approach, they further analyzed the lung phenotype of Sp3-/- vs. Sp3+/+ (WT) embryos at E15,

© 2023. Published by The Company of Biologists under the terms of the Creative Commons Attribution License (https://creativecommons.org/licenses/by/4.0/).

E18 and immediately after birth. They used bulk RNAseq to tease out the transcriptomic changes in the lung mesenchyme at E15. They also provide evidence for impaired lung vasculature, decreased mesenchymal proliferation, abnormal myofibroblast differentiation and increased lipofibroblast formation.

In general, the paper is well written and the results are convincing.

Major critiques

1_ It would be informative to quantify the expression of Sp3 in the epithelium and mesenchyme during lung development over time to give some context to the work. Is Sp3 expressed at higher level in the mesenchyme? In which mesenchymal clusters? The use of available data set (scRNAseq or bulk RNAseq) should render this feasible and cost effective.

We previously used confocal immunomicroscopy to demonstrate Sp1 and Sp3 expression in developing mouse lung tissue from E13 to postnatal day 1. We did not observe significant changes in expression over time and expression also appeared relatively evenly distributed. We now include reference to that data. In addition, we have included Sp3 expression data from another previously published scRNAseq dataset, again showing Sp3 expression throughout all cell populations (Supplemental Figure S1).

2_ Figure 1: the comparison of the branching process at E15 between WT and KO clearly indicates a branching defect already. The authors should quantify this. In addition, it may be informative to look at earlier time points (E12.5) to see if the defects are already present. This would give the authors the opportunity to look for the classical genes involved in the branching process (Fgf10, Fgfr2b, Etv4/5, Shh, etc...). Is Sp3 involved in the control of these canonical genes regulating the branching process? The heatmap in figure 4B (looking at developmental signaling is very informative for the branching process as it shows reduced Fgf10 expression at E15.5. Does this translate into less proliferation in the lung epithelium at E15.5? The authors should also look at markers of epithelial differentiation (proximal and distal markers of differentiation). This would make their paper very informative. Is the epithelium just paying the price of mesenchymal defects due to Sp3 deletion in the mesenchyme or are they cell-autonomous defects in the epithelium?

To characterize earlier branching morphogenesis, we isolated fetal lungs at E12 and quantified their subsequent branching by live tissue microscopy. These data are now included in Figure 1. There did appear to be some early differences in branching morphogenesis between WT and $Sp3^{-/-}$ lungs, with the differences becoming more obvious over time.

In addition, we include real time PCR data from total lung RNA comparing the expression of multiple transcripts important for branching morphogenesis in Supplemental Figures 2 and 3. While these data did not show a clear difference based on genotype, future experiments will hopefully be able to investigate these differences at higher resolution.

3_ What is the significance of increased Axin2 expression in Sp3-/- mesenchyme? Is more Wnt signaling (which needs to be demonstrated) consistent with the phenotypes described in terms of proliferation and differentiation?

We include additional data using total lung RNA comparing expression of multiple transcripts in the Wnt signaling pathway. The data did not show a clear difference that allowed us to identify a molecular connection between Sp3 and the Wnt pathway. Therefore, we believe that additional future studies may be required to determine if the increase in Axin2 in primary $Sp3^{-/-}$ mesenchymal cells is cell autonomous. We have included changes in the text discussing the potential role of Wnt signaling in mesenchymal cell proliferation and/or differentiation.

4_ Figure 3B: for the heatmap, the authors should first show the Wild type and then the Sp3ko samples (like for the heatmaps above and underneath).

We have revised all figures for consistency.

© 2023. Published by The Company of Biologists under the terms of the Creative Commons Attribution License (https://creativecommons.org/licenses/by/4.0/).

5_ The increase in Lipofibroblast number is Sp3ko lungs is very puzzling as the authors show also a decrease in Fgf10 expression (Figure 4B). Could this be due to decreased TGFb signaling (Tgfb signaling has also been proposed to prevent LIF formation). It would also be interesting to show if the mesenchyme from Sp3Ko lungs could be more supportive for organoid formation (use of postnatal AT2s may be possible here but would need to be established). A general question from this reviewer is: what are the consequences of increased LIF on the AT2s?

The reviewer raises a very intriguing question about the potential connections between Sp3 and TGFβ signaling. We have included additional data showing expression of TGFβ ligands and receptors in WT and $Sp3^{-/-}$ lungs, without obvious differences. Given the complexity of TGFβ signaling within tissue niches, we recognize that more in depth studies would be required to fully investigate this potential mechanism. We have also included additional text to discuss the potential role of Sp3-dependent regulation of TGFβ in regulating the myofibroblast/lipofibroblast phenotype.

Regarding AT2 cell number, we have included additional data in Figure 7 showing AT1 and AT2 differentiation in WT and $Sp3^{-/-}$ developing lungs. There was not an obvious difference in AT2 cell differentiation in $Sp3^{-/-}$ lungs.

6_ It is not clear why the authors do not show directly proliferation in the lung mesenchyme in situ (Figure 8A-C: they show instead the liver). An explanation should at least be given for this choice. Why did the authors feel the need to immortalize the lung mesenchyme cells?

Our initial experiments detected very low proliferation rates within the embryonal lung mesenchyme. We therefore measured proliferation in the fetal liver, which was reduced in $Sp3^{-/-}$ tissues. In addition, Sp3 deletion had a greater impact on CyclinE expression in liver compared to lung. We clarify these points in the text and discuss potential explanations. Conditionally immortalizing the Sp3 mutant cells allowed expansion and passaging in culture, which was required for studying cell cycle progression. We clarify this rationale in our revision.

7_ Unless I missed it, it seems that the bulk RNAseq analysis was done only at E15.5, it would be interesting to do it also at E18.5 and P0. If it is possible to generate more K0 embryos, it would be great to quantify the defect in the epithelium and mesenchyme by FACS to quantify the abundance of different subpopulations.

The reviewer is correct that our RNAseq studies used fetal lung mesenchymal cells isolated from E15 mice. We have included gene expression data from E15, E18, and P0 lungs in our revised manuscript. We have also included FACS data showing a slight increase in $CD31^{+}$ cells with a subsequent reduction in lineage-negative (mesenchymal) populations in P0 $Sp3^{-/-}$ lungs. No differences in epithelial abundance were detected. Interestingly, our RNAseq data also measured increased *cd34* and *vwf* expression in $Sp3^{-/-}$ mesenchymal cells, suggesting that Sp3 may have additional roles in regulating mesenchymal cell differentiation.

Reviewer 2 Advance Summary and Potential Significance to Field:
In this manuscript, McCoy et al. used Sp3-/- mouse embryos and cultured lung mesenchymal cells to demonstrate that SP3 transcription factor is essential for normal lung morphogenesis and cell cycle progression during mouse embryonic development. RNAseq and cellular phenotyping in Sp3-/- embryos identified changes in extracellular matrix deposition, cell proliferation, and fibroblast differentiation which resulted in multiple structural defects, including lung vascular abnormalities, lack of elastic fiber formation and accumulation of lipids within lung fibroblasts. Based on in vitro and in vivo data, Sp3-/- cells exhibited cell cycle arrest with accumulation of cells in G0/G1 phase of the cell cycle, which was due to decreased expression of multiple cell cycle regulators, including Cyclin E, implicating SP3 in activation of Cyclin E expression. Overall, the present study convincingly demonstrates the global impact of SP3 on lung embryonic development in the mouse. The manuscript contains novel and high-quality experimental data, and main conclusions are supported by experimental data, identifying SP3 as a novel regulator of lung development.

Reviewer 2 Comments for the Author:
Several points need to be addressed to improve overall quality if the manuscript:

© 2023. Published by The Company of Biologists under the terms of the Creative Commons Attribution License (https://creativecommons.org/licenses/by/4.0/).

Specific points:

1.It is unclear what is the rationale for brief culturing of lung mesenchymal cells prior to RNAseq. Is this for purification purposes? The authors should consider and discuss a possibility that cell culture conditions can change gene expression in lung mesenchyme.

We absolutely recognize that cell culture conditions can cause transcriptional changes. By passaging mesenchymal cells, we obtained sufficient cell numbers and reduced other cell populations within the cultures. We have clarified these points and important caveats in our revised manuscript.

2.Vascular changes and the capillary density in E15 lungs should be quantitated, preferably, in 3D.

We have included new images from E15, E18, and PND0 lungs in our revised manuscript. Figure 5 also now includes 3-D maximum projections of confocal sections obtained using thicker tissue slices. These data better illustrate the differences in cell shape seen in $Sp3^{-/-}$ lung endothelial cells, especially at earlier time points.

3.In Fig. 5D, the authors refer to “elongated cellular processes”. This should be corrected. Low magnification images show only the location of endothelial cells and basic structure of the capillary network. The endothelial cell processes cannot be clearly seen at these low magnification images. Additional high magnification images are needed in Fig. 5.

We include higher magnification images in the revised Figure 5 along with revising the text to more accurately describe the observed phenotype.

4.Western blot in Fig. 9E clearly shows a decrease in Cyclin E protein in E15 Sp3-/-lungs. However, quantification of these data does not indicate statistically significant differences in E15 Sp3-/-lungs because the blot looks over-exposed. This leads to overall impression that the quantification is not consistent with Western blot images.

While Cyclin E expression in $Sp3^{-/-}$ lungs was slightly lower than in WT lungs, the difference did not reach statistical significance. We have clarified this finding in the revised manuscript and have now included datapoints for each replicate in Figure 9.

5.It is unclear whether SP3 regulates Cyclin E expression through direct (transcriptional) or indirect mechanisms.

While we are still designing approaches to address this question, the *Ccne1* promoter/enhancers contain multiple Sp protein binding sites. We have included a graphical representation of these regions from publicly available ChIPseq data in Figure 9.

Reviewer 3 Advance Summary and Potential Significance to Field:
This manuscript by McCoy et al. describes the effect of deleting Sp3 on mouse lung development. The authors show that deletion of Sp3 impairs vascular and mesenchymal development in the mouse lung. Imbalance in myofibroblast/lipofibrobalsts differentiation as well as defects in vascular development are seen following Sp3-/-. Detailed transcriptional profiling and thorough analyses of Sp3-/- fetal lung mesenchymal cells provides insight into the genes regulated by Sp3.

Reviewer 3 Comments for the Author:
This manuscript by McCoy et al. describes the effect of deleting Sp3 on mouse lung development. While the study is very descriptive, the experiments were carefully carried out and the findings include detailed transcriptional profiling and validation of genes and pathways altered by the absence of Sp3. While the study is interesting, several issues need to be addressed by the authors.

Major:

Was there a difference in lung size at E15? Since the authors show branching defects in cultured explants, it would be interesting to know at what stage the lung defects start? And whether there is overall less branches at E12 and/or E14?

© 2023. Published by The Company of Biologists under the terms of the Creative Commons Attribution License (https://creativecommons.org/licenses/by/4.0/).

As mentioned in our response to reviewer 1, we have now included additional data in Figure 1. There did appear to be a reduction in airway branching at earlier stages of development in Sp3$^{-/-}$ lungs.

Authors claim that Sp3-/- explants “displayed thicker interstitial regions”. H7E to support this claim should be provided in Figure 1.

We have included additional data in Figure 1H showing higher magnification images of representative explants that illustrate the mesenchymal differences. Quantitative data are also now included in Figure 1I.

Authors should provide additional details on passage number for the mesenchymal cells that were transcriptionally profiled. What is “brief culture period”? were these cells treated with IFN-g in culture?

Freshly isolated cells were cultured for 2 passages before being used for RNAseq. IFN-gamma was only used in the conditionally immortalized cells in the cell cycle experiments in Figures 8 and 9. We have clarified these details in our revised manuscript.

Page 9, line 174. It would be helpful if the authors cited in the results section examples of those differentially expressed genes that are markers of lipo- and myo- fibroblasts phenotypes. Alternatively, a more detailed description of results and data in Figure 4 could be provided.

We have included a more detailed discussion of these data in the revised manuscript.

The authors should include either an excel sheet detailing all the differentially expressed genes or make their sequencing data available online.

All gene expression data will be made available through the Gene Expression Omnibus upon manuscript acceptance.

Figure 6: The claim of rounder a-SMA+ cells isn’t convincing based on the figures shown. In addition, what is the difference between panels B and C and panels E and F?

We have included additional confocal images in our revised Figure 6 along with 3-D maximum projections of α-SMA^{+} cells. The α-SMA^{+} cells in Sp3$^{-/-}$ lungs appeared to express α-SMA in a more cytoplasmic, g-actin pattern with reduced stress fiber formation. Interestingly, smooth muscle cells around vessels and large airways appeared similar. We have revised the text to describe the observed differences more accurately

The rationale for staining liver sections for cell proliferation in Figure 8 isn’t clear. The authors have lung sections available, why did they not stain lung sections?

As mentioned above for the comments raised by Reviewer 1, cell proliferation by our measurement was low in the lung, making quantification of lung cell proliferation challenging. We have clarified this point in our revised manuscript.

Minor:

Please include Scale bars for Figure 1D

Figure 5 B and D, the nuclear staining should be improved. Scale bar size should be verified. Panel D should be replaced with a similar region/area as to what is shown in panel A

Figure 6 panel J: please provide a higher resolution image

We have included new images and scale bars for each figure.

© 2023. Published by The Company of Biologists under the terms of the Creative Commons Attribution License (https://creativecommons.org/licenses/by/4.0/).

Second decision letter

MS ID#: DEVELOP/2022/200839

MS TITLE: Sp3 Is Essential for Normal Lung Morphogenesis and Cell Cycle Progression During Mouse Embryonic Development

AUTHORS: Alyssa M. McCoy, Omar Lakhdari, Sayane Shome, Kaitlin Caoili, Gilberto E E. Hernandez, Nima Aghaeepour, Lindsay D. Butcher, Kathleen Fisch, and Lawrence S. Prince
ARTICLE TYPE: Research Article

I am happy to tell you that your manuscript has been accepted for publication in Development, pending our standard ethics checks.

Reviewer 3

Advance summary and potential significance to field

The authors have adequately addressed all previous critiques and comments by reviewers. this reviewer has no further concerns.

Comments for the author

The authors have adequately addressed all previous critiques and comments by reviewers. this reviewer has no further concerns.

© 2023. Published by The Company of Biologists under the terms of the Creative Commons Attribution License (https://creativecommons.org/licenses/by/4.0/).
